# Ion-Conducting Flexible Thin Films of Composites from Poly(ethylene oxide) and Nematic Liquid Crystals E8—Characterization by Impedance and Dielectric Relaxation Spectroscopy

**DOI:** 10.3390/polym13244465

**Published:** 2021-12-20

**Authors:** Georgi B. Hadjichristov, Todor E. Vlakhov, Yordan G. Marinov, Nicola Scaramuzza

**Affiliations:** 1 Georgi Nadjakov Institute of Solid State Physics, Bulgarian Academy of Sciences, 72 Tzarigradsko Chaussee Blvd., 1784 Sofia, Bulgaria; todor_vlakhov@issp.bas.bg (T.E.V.); ymarinov@issp.bas.bg (Y.G.M.); 2 Dipartimento di Fisica, Università degli Studi della Calabria (UNICAL), Via P. Bucci, Cubo 33B, 87036 Rende, Italy; nicola.scaramuzza@fis.unical.it

**Keywords:** polymer–liquid crystal composites, electrolytes, alkali metal ion-polymer complexes, electrical properties, ionic conductivity, dielectric properties, complex electrical impedance spectroscopy, complex dielectric spectroscopy, relaxation processes

## Abstract

Complex electrical impedance and dielectric spectroscopy were applied to study the dielectric relaxations and their thermal behavior in ion-conducting composites/complexes from polymer poly(ethylene oxide) (PEO) and E8 nematic liquid crystals (LCs), at the compositional ratio PEO:E8 = 70:30 wt%. Flexible thin films of PEO/E8 with a thickness of 150 μm were inspected, as well as such films from Na^+^ ion-conducting electrolyte PEO/E8/NaIO_4_ with the same PEO:E8 compositional ratio, but additionally containing 10 wt.% from the salt sodium metaperiodate (NaIO_4_) as a dopant of Na^+^ ions. The molecular dynamics, namely the dielectric relaxation of PEO/E8 and PEO/E8/NaIO_4_, were characterized through analyses of complex impedance and dielectric spectra measured in the frequency range of 1 Hz–1 MHz, under variation of temperature from below to above the glass-transition temperature of these composites. The relaxation and polarization of dipole formations in PEO/E8 and PEO/E8/NaIO_4_ were evidenced and compared in terms of both electrical impedance and dielectric response depending on temperature. The results obtained for molecular organization, molecular relaxation dynamics, and electric polarization in the studied ion-conducting polymer/LC composites/complexes can be helpful in the optimization of their structure and performance, and are attractive for applications in flexible organic electronics, energy storage devices, and mechatronics.

## 1. Introduction

Solid-state and flexible composites from polymers and liquid crystals (LCs) are multifunctional materials with great potential and are of current interest for advanced applications, e.g., for the realization of flexible displays and smart windows [1,2,3,4,5,6]. Having suitable mechanical, electrical, dielectric, thermal, and other properties, as well as relatively easy preparation, stable chemical properties, and film-forming behaviors, and providing high mobility of charge carriers, such all-organic soft-solid composite materials can be ionic conductors that combine the advantages of solid polymer electrolytes with the unique properties of the LC soft matter included in plastic materials. For this reason, promising polymer-LC combinations are developed for alternative electrical energy storage systems, for thin-film devices, such as dry-state mini-batteries, electromechanical actuators, solar cells, solar-energy harvesting, and electrochromic displays, as well as for sensorics, mechatronics, and soft electronics applications [7,8,9,10,11,12,13,14,15].

The synthetic polymer poly(ethylene oxide) (PEO) is often used as a polymer matrix in polymer–salt composites’ electrolytes [16,17,18]. PEO is a semi-crystalline material with flexible ethylene oxide segments [19]. At ambient temperature, PEO exhibits sufficient chain flexibility, and the semi-crystalline structure of this polymer makes it suitable as a host for alkali metal ions and supports ionic conductivity [16,17,18]. PEO-based polymer electrolytes are among the most promising materials due to their good thermal properties and interfacial stability with metal electrodes [20,21,22,23,24,25,26,27,28,29,30]. The low glass transition temperature (*T_g_*) of PEO, as well as its flexibility and other valuable properties, are well suited for engineering composite materials made from PEO and mesogens.

The research interest in polymer-LC materials is driven by the possibility of forming flexible electrolyte membranes with enhanced response and functionality. Due to the improved electrical properties, various electrolytic polymer-LCs molecular systems based on polymer PEO have been synthesized and investigated [31,32,33,34]. New fields of development and application of such polymer electrolytic materials are challenging scientific research areas, and many studies are focused on this. In particular, novel electrolyte materials with microscopic porosity are attractive for fundamental research and industrial applications [35]. Previously, flexible polymer/LC composites from PEO and the LC mixture with the commercial name E8 were synthesized, and thin films of PEO/E8LC were investigated [36]. The well-known room-temperature nematic E8 is a eutectic mixture of LC cyanobiphenyl derivatives and cyanoterphenyl LCs. E8LC is characterized by high chemical and thermal stability, a wide temperature range of the nematic phase, relatively large dielectric anisotropy, as well as proper elastic constants and viscosity [37].

PEO/E8 composites have great potential as a platform for producing electrolytes and other functional materials for sensorics and organic electronics applications. Compared to pure PEO, these composites were found to have considerably enhanced electrical conductivity and dielectric permittivity, which can be tailored by their phase-separated morphology [36]. Further, PEO/E8/NaIO_4_ Na^+^ ion-conducting polymer-LC electrolytes were synthesized and examined [38]—they demonstrated excellent ion electrolytic and dielectric properties [38,39], and hence, are attractive for diverse applications. In the PEO/E8 and PEO/E8/NaIO_4_ ion conductors, an intermolecular coupling between PEO and E8LC was suggested [39]. In the present study, the frequency spectra of complex electrical impedance and dielectric permittivity of thin films of these composites were analyzed depending on the temperature in the vicinity of their glass transition—a situation in which the dipole contribution to impedance and dielectric response can be well sensed. The study of dipole polarization and dielectric relaxations in the inspected PEO/E8-based ion-conducting polymer/LC composites and their temperature dependencies is directly related to the practical applications of the thin films of these advanced materials. To elucidate on the role of the embedded LCs, the temperature-dependent dielectric properties of both PEO/E8 and PEO/E8/NaIO_4_ were compared.

## 2. Materials and Methods

Details regarding the materials, preparation of samples, and experimental procedures using complex impedance spectroscopy are given elsewhere [36,38] but are reported briefly in this section. Polymer/LC composites were produced from PEO and LC E8 (from Merk) (Figure S1a, Supplementary Materials) via their mixing at a ratio PEO:E8 = 70:30 by weight (wt.%). This ratio was found to be optimal for both the ion conductivity and dielectric properties of these composites [36]. To produce PEO/E8/NaIO_4_ ion-polymer complexes, the salt sodium metaperiodate (NaIO_4_) was added at a concentration of 10 wt.% to the PEO/E8 composite; the compositional ratio was PEO:E8:NaIO_4_ = 63:27:10 wt.%. Such content of NaIO_4_ ensured a fairly good ion conductivity and suitable dielectric permittivity of the PEO/E8/NaIO_4_ electrolyte films without changing the other properties, quality, and long-term stability [38]. Free-standing, flexible thin films with a thickness of 150 µm of both PEO/E8 and PEO/PVP/NaIO_4_ were prepared by the conventional solution cast technique. As in our previous studies [36,38], the structural analyses by XRD, polarization optical microscopy, FTIR, and micro-Raman spectroscopy evidenced the successful synthesis of these complex molecular formations and the homogeneous dispersion of E8 LC into the PEO polymer. Mixed polymer and LC phases in these composites, as well as LC micro-droplet structures, were clearly displayed by optical microscopy [39] (Figure S2). The produced flexible thin films were sandwiched between two flat glass plates covered with ultrathin (~80 nm) conductive layers of indium tin oxide (ITO) (from Delta Technologies Ltd., Loveland, CO, USA) that served as electrodes with low (<10 Ω/sq) sheet resistivity. Thus, the electrical contact occurred via the ITO coating area, *A* = 1 cm^2^ (a square with a size *s* = 10 mm ± 0.1 mm). The thickness of the poly/LC composite films was measured with a Coolant IP 65 digital micrometer (Mitutoyo Co., Takatsu-ku, Kawasaki, Kanagawa, Japan) with an accuracy of ±1 µm. For the sake of comparison, reference films of E8LC with thicknesses of 25 µm were also prepared in glass cells (KSRO-25/B111N1NSS Up/Low, manufactured by E.H.C. Co. Ltd., Hachioji, Tokyo, Japan). The electrically active areas of these cells (their inner surfaces coated with ITO layers) were also 1 cm × 1 cm.

The frequency spectra of the complex electrical impedance (Z*) of the polymer/LC composite films were measured using the SP–200 high-precision impedentiometric workstation (Bio-Logic Science Instruments, Seyssinet-Pariset, Grenoble, France). They were recorded over the frequency range from 1 Hz to 1 MHz of the applied alternating-current (AC) electric field. AC voltage in the sine waveform was applied transversally to the films. The measurements were performed at an amplitude of 0.1 V_RMS_. According to specifications of the SP-200 instrument, the relative error by measurement of electrical impedances in the range below 3 MHz did not exceed 1% (below 500 kHz, the performance was higher and reached 0.3%). The measurements of all samples were made under identical experimental conditions. The time interval between data acquisition of experimental points during the frequency scans was kept the same.

The value of ion conductivity (σ) of the studied composite films was estimated by the expression σ = *d*/(*R_B_ A*), where *d*, *R_B_* and *A* represent the thickness, bulk resistance, and electrically active area of the sample (the contact area of the electrodes), respectively. The *R_B_* of the films was obtained from the Nyquist complex impedance diagrams. The accuracy of the calculated values of σ was restricted by the uncertainty of geometric parameters—the measured thickness *d* of the films and the electrode sizes (*s*). These uncertainties were *δd* = ±1 µm and *δs* = ±0.1 mm, resulting in an overall uncertainty of ±3.6% for the values of σ, taking into account the relative error of 1% (maximum) for the measured value of the resistance *R_B_*.

The temperature of the samples was maintained using a Mettler FP82 hot stage (thermostat) interfaced with a computer. The temperature was stabilized and measured to within ±0.1 °C. Complex impedance spectra were recorded one after another in sequence, at temperature intervals of ca. 2 °C, in the range of 22 °C to 68 °C. At every fixed temperature value, the samples were kept for 10 min to obtain thermal equilibrium before the measurement of impedance. The fits to the measured dielectric data were performed using a self-made program based on code from the LEVMW v.8.11 complex nonlinear least-squares fitting and inversion program.

## 3. Results and Discussion

### 3.1. Impedance Spectra Analysis

Temperature-dependent frequency spectra of the complex electrical impedance (*Z*) for both PEO/E8 and PEO/E8/NaIO_4_ composite films were obtained under identical experimental conditions by varying the temperature (*T*) from ambient (room) temperature, *T_room_*, to values slightly above their melting temperatures (*T_m_*). By differential scanning calorimetry (DSC), we measured *T_m_* = 62.3 °C for PEO/E8 and *T_m_* = 57.3 °C for PEO/E8/NaIO_4_ (Figure S3). As determined from DSC scans, the glass transition temperatures (*T_g_*) of both composites were *T_g_*(PEO/E8) = 52 °C and *T_g_* (PEO/E8/NaIO_4_) = 42.5 °C (Figure S3). Being a combination of a soft fluid-like LC material E8 and polymer PEO, at *T_room_*, these composites were in their ‘glassy’-like state. Note that *T_g_*(PEO) = −52 °C; *T_m_*(PEO) = 70.3 °C; *T_g_*(E8) = −98 °C [40], as well as the temperature of the phase transition from the nematic to isotropic phase for E8LC (*T_N-I_* = 72 °C) [41], were beyond the temperature interval from *T_room_* to *T_m_*.

**Figure 1:**
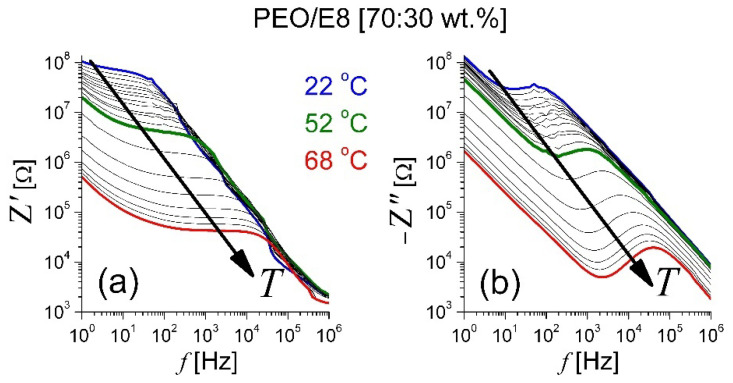
Frequency spectra of complex electrical impedance of PEO/E8 composite film: (**a**) real part (*Z′*) and (**b**) imaginary part (*Z″*). Spectra were recorded under identical experimental conditions, by varying the temperature of the film in the range of 22 °C to 68 °C. The arrows show the effect of increasing temperature.

Figure 1 reports the couples of frequency spectra {*Z′*, *Z″*} of the real (*Z′*) and imaginary (*Z″*) parts of Z* of the PEO/E8 composite film, simultaneously measured as a function of the frequency *f* of the AC electric field applied to the film at gradually elevating temperature in twenty equal steps within the range of 22–68 °C. The impedance behaviors were relaxational. In particular, each of the *Z″*(*f*) spectra displayed a pair of a broad minimum (at lower *f*) and one broad peak (at higher *f*), both related to typical dielectric relaxation in dielectric media [42,43,44,45,46,47,48]. It is known that the minimum in *Z″*(*f*) spectrum is related to the electrode polarization effect, i.e., the accumulation of charges in the electrode-electrolyte interface and the formation of an electric double-layer [46,47,49,50]. The presence of a double-layer (electrode polarization) strongly affects the observation of the dielectric relaxation processes. In general, the relaxation phenomena are controlled by free-volume-activated kinetics or by thermally activated kinetics.

The maximum of *Z″* corresponded to the main dielectric-active relaxation that occurred in the dielectric materials under the action of an external AC electric field (i.e., by induced electric polarization). The frequency of the *Z″* peak (*f_maxZ″_*), known as the relaxation frequency, defined the characteristic relaxation time τ = (2π *f_maxZ″_*)^–1^ of the medium. In general, in such media studied by impedance spectroscopy, one can accept that this peak originates from the orientation of dipoles (dielectric relaxation of the orientational electric dipole polarization). From Figure 1b, it is possible to observe that the gradually increased temperature of the studied polymer/LC composite led to a shift of *f_maxZ″_* (together with the frequency of the minimum in the *Z″* spectra) towards higher frequencies, from 50 Hz (at 22 °C) to 40 kHz (at 68 °C). The observed shift resulted from dielectric relaxation and was due to the decrease in the dielectric relaxation time [42,43,44,45,48]. By comparison with the *Z* spectra of the two ingredients of the PEO/E8 composite—PEO and E8LC—measured under the same experimental conditions (Figure S4), it is clear that the effect from the embedded E8 LC molecules was significant and resulted in impedimetric and dielectric relaxation behaviors that were intermediate between those of the polymer and the LC. 

The {*Z′*, *Z″*} spectra and dielectric relaxation behaviors of the PEO/E8 composite, as well as their temperature change, suggested a dipolar molecular organization in this polymer/LC system. In our case, the dielectric relaxation may have been a typical dipole reorganization in the PEO/E8 composite caused by applying an external AC electric field. The relaxation in the dielectric PEO/E8 upon the AC field was related to the contributions of both the polymer chains of PEO and the LC soft matter. Coupled molecular dipoles are possible in such polymer/LC composites. Intermolecular PEO-E8LC formations owing to functional groups of both PEO and E8 LC have been evidenced by microstructural investigations of the studied PEO/E8 composite [36,39]. PEO-E8LC intermolecular coupling may have occurred due to electric dipole–dipole interaction between the LC molecules of E8 and PEO oxygen in the structural units of PEO (Figure S1b). Cyano compounds in E8LC promoted PEO-E8LC interactions since they had highly polarized cyano groups and easily polarizable biphenyl groups (Figure S1a). The cyano end group in the chemical structures of the E8LC molecules was strongly electron-withdrawing, while the oxygen atoms in the [–CH_2_–O–CH_2_]_n_ backbone structure of PEO were electron-donating. The C-O-C bond in the ether functional group of PEO was polar (slightly polar). The polar molecular configurations in PEO/E8LC composite can be considered as coupled dipoles (Figure S1b) formed from single or more LC molecules of nematic E8 with ether oxygens in the C-O-C subunit of PEO, in the process of synthesis of the composite. The finite relative displacement of positive and negative charges produced composite dipolar molecular formations in PEO/E8LC throughout a given spatial volume in which these charges were present. The strong interactions between PEO polymer chains and dispersed rod-like molecules of E8LC led, in practical terms, to a coupling of the LC molecules to the polymer backbone, and their electric-field-driven reorientation was strongly hampered. Due to the flexoelectric and flexo-dielectric properties of nematic LC molecules, the coupled PEO-E8LC molecular dipoles can be further considered flexo-dipoles. The impedance data discussed above, and the following results, support such a model of dipolar molecular organization and dipole–dipole interaction in the studied PEO/E8 composite.

The dipole properties of the LC component in the investigated polymer/LC composites should be considered, especially in the low-frequency region. The polar E8 LC molecules that ‘decorate’ the PEO polymer network (the molecules being coupled to the polymer chains) strongly contributed to the dielectric relaxation of PEO/E8 at frequencies that tended to zero. In this case, the well-pronounced increase in *Z″* values can be attributed to a ‘soft mode’ relaxation. Such an effect was previously observed in nematic LCs with high dielectric anisotropy (rod-like LC molecules with large dipole moments, similar to the nematic LCs in the E8 LC mixture employed here [37]) when confined in micropores [51]. In our case, the composite film composed of 70 wt.% PEO and 30 wt.% E8LC can be considered a 3D spongy membrane (network of the polymer matrix) in which nematic LCs permeate the pores of the polymer (LC molecules embedded in a porous network). The microscopic porosity of the studied composites (pores and microvoids, probably interconnected) was evidenced by scanning electron microscopy (SEM) studies [52]. The ‘soft mode’ dielectric relaxation is well established for polar nematic LCs, such as pentylcyanobiphenyl (5CB), filling micropores [51]. The nematic LC mixture E8 contained cyanophenyl LC molecules that were polar. In particular, 5CB, the main ingredient of the E8 LC mixture, was found to be strongly polar (the permanent dipole moment of the 5CB molecule is ~5 D). Being coupled with the polymer chains in PEO/E8, the polar nematic LC molecules interacted with the polymer network through their surface. Most probably, the dipole–dipole (electrostatic) interaction between confined dipole LC molecules was strongly suppressed due to their strong interaction with the polymer network. Together with the polymer backbone, the LC dipoles took part in the polymer chain motion, in the thermodynamical behavior, as well as in any thermal fluctuations of the polymer-LC composite considered here.

It must be noted that the increase in *Z″* at frequencies that tended to zero also took place in the *Z″* spectrum of pure PEO (Figure S4), but this effect was due to electrode polarization only (play the ions inherent for PEO). ‘Free’ charges are always present in polymeric materials, in particular polymer films prepared using the solution-cast technique. In pure PEO, as reported in the literature, the presence of ‘free charge’ increases the dielectric losses by decreasing frequencies. At the low-frequency limit, the values of *Z″* for PEO do not correspond to the bulk dielectric function but are somewhat due to the ‘free’ charge of conducting species build-up at the interface between the material and the electrodes. When preparing the composite of PEO and the nematic LC E8, the ‘free’ charges remain “frozen” in the polymer structure, and the answer is that of the nematic LC with a large electric dipole. The effect on the impedance is similar—increased *Z″* at frequencies that tend to zero—but the phenomenon is different. It is seen in Figure S4 that, while for pure PEO after the local maximum of *Z″*, the reduction in *Z″* at decreasing *f* was small because the ‘free’ charges came into play; in the case of the PEO/E8 film, there was a significant decrease in the *Z″* values up to a well-defined minimum. Then, an increase occurred when the ‘soft mode’ relaxation of the LCs with sizable electric dipole came into play, and the dielectric losses increased again.

It is noted in Figure 1 that the frequency *f_soft_*, at which the ‘soft mode’ was triggered, did increase at increasing temperatures. This agrees with theory as the characteristic time of the ‘soft mode’ in nematic LC is directly proportional to the rotational viscosity, decreasing with increasing temperature [51]. Near *T_g_*, the frequency *f_soft_* approached the dielectric relaxation frequency of pure LC E8 (Figure S4). At *T* > *T_g_*, the ‘soft mode’ was well pronounced. With the addition of sodium salt to the PEO/E8 composite, everything became more complicated, but in any case, these phenomena were present in a specific temperature range.

**Figure 2:**
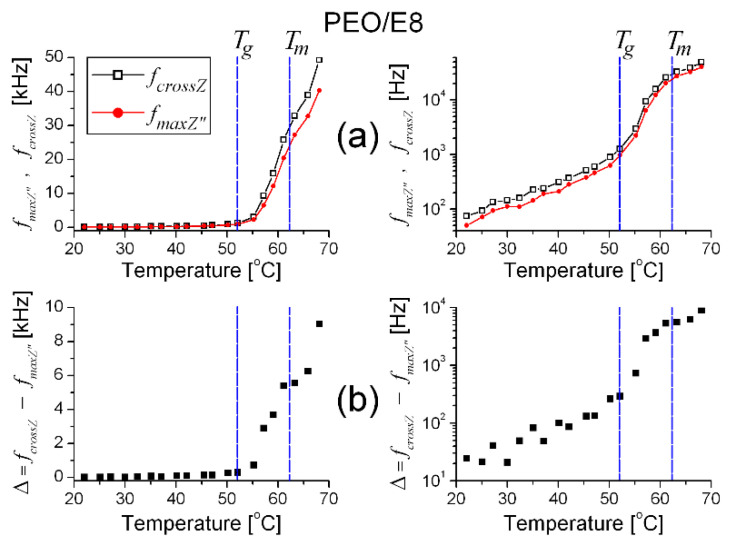
The frequencies *f_cross_* and *f_maxZ″_* (**a**), as well as the difference Δ = *f_cross_* − *f_maxZ″_* (**b**) vs. the temperature for PEO/E8 composite, in linear (**left**) and logarithmic (**right**) scale. The dashed lines point out the glass transition (*T_g_*) and melting temperature (*T_m_*) values of the PEO/E8 composite. The uncertainty limits for the experimental data presented are within the size of the symbols for the data.

Besides *f_maxZ”_*, the complex *Z* spectra of the materials can be also characterized with the cross-frequency (*f_crossZ_*) at which *Z′* = *Z″* (Figure S5). Figure 2 shows the temperature-dependent variation in both *f_maxZ″_* and *f_crossZ_*, as well as their difference Δ = *f_crossZ_* − *f_maxZ″_*, for the studied PEO/E8 polymer/LC composite. Because these characteristics have some peculiarities, the frequency values are presented in linear, as well as in logarithmic scales. The results from the analysis of these dependencies can be summarized as follows:

(i) *f_crossZ_* > *f_maxZ″_* in the measured range of temperature (*T*), from *T_room_* to the temperature slightly above the melting point *T_m_* (Figure 2a) when an utterly amorphous phase of PEO is present (amorphous rubbery state);
(ii) Both *f_crossZ_* and *f_maxZ″_* were increasing functions of *T* (Figure 2a);
(iii) In the temperature dependencies of both *f_crossZ_* and *f_maxZ″_*, there was a distinct threshold (*T_th_*)—above the temperature *T_th_* up to *T_m_*, these dependencies were enhanced (at *T* > *T_m_* the increment was lowered); the threshold *T_th_* was equal for both *f_crossZ_* and *f_maxZ″_*, and *T_th_* ≡ *T_g_* (Figure 2a);
(iv) The difference Δ = *f_crossZ_* − *f_maxZ″_* was also an increasing function of *T*; Δ was steeply enlarged above the same temperature threshold *T_th_* ≡ *T_g_* (Figure 2b).

The accuracy of the measured values of temperature, the frequency (*f_crossZ_* and *f_maxZ″_*) and their difference Δ was: ±0.1 °C; ±0.1 Hz and ±0.2 Hz, respectively. Thus, the sizes of the error bars (both *xErr* and *yErr*) for the experimental data presented in Figure 2 should be much smaller than the size of the data symbols in the graphs (in both linear and log scales).

It should be emphasized that the relation *f_crossZ_* = *f_maxZ″_* holds for the ideal (dipolar) dielectric. This case corresponds to perfect Debye relaxation—a relaxation of non-interacting dipoles. Differentiating *f_crossZ_* and *f_maxZ″_* means a deviation from the ideal case, and *f_crossZ_* > *f_maxZ″_* is the usual relation, in particular for PEO-based ion-conducting systems [53,54]. The increase in Δ with the increasing *T* indicates a progressive dispersion of the dielectric relaxation peak, a deviation from the ideal dipolar dielectric (for an ideal dielectric, the peak should be a Dirac delta instead of a bell-like curve) and is a sign of dipole–dipole relaxation. As a result of heating the PEO/E8 composite, all these features were enhanced, especially in the temperature range *T_g_* < *T* < *T_m_*. The above observations (ii)–(iv) imply a typical effect due to dipole relaxation. Such thermal behaviors of *f_maxZ″_* and Δ can be associated with interacting dipoles and dipole relaxation, which became more intensive because of the softening of the polymer at *T* > *T_g_*. Therefore, these results support the model of dipolar molecular organization in the considered polymer/LC composite and the dipolar character of the molecular interaction (dipole–dipole interaction), as well as the above assumption on the possibility for polar PEO-E8LC intermolecular formations.

As known, at *T_g_*, a specific softening of the polymers and plastics occurs, and the random moving of the polymer chains is intensified, known as long-range segmental motion. Below *T_g_*, the plastics behave as rigid glasses (glassy state). On heating of the polymer above *T_g_*, the polymer chains become freer to move. The proportion of free volume increases until there is a sufficient free volume for large-scale movements of the flexible polymer backbone, and the polymer does change from being hard to being soft and more flexible. By polymeric materials, the functional relationship between *f_maxZ″_* and *T* is continuous through the melting point, and *f_maxZ″_*(*T*) is also increasing but at a smaller slope, as reported, in particular, for PEO [55]. In our case, *T_m_*(PEO/E8LC) was lower (by 8 °C) than *T_m_*(PEO) (Figure S3), indicating a significant structural change and an enhanced polymer chain flexibility—this was due to embedded mesogenic LCs at the concentration 30 wt.%. This happens during the synthesis of these PEO-based composite systems. The inclusion of NaIO_4_ salt additionally reduced the *T_g_* and *T_m_* of the considered composites by ca.10 °C and 5 °C, respectively (Figure S3). As assessed by DSC, microstructural, and optical spectroscopy studies [38], the addition of 10 wt.% NaIO_4_ leads to a considerable increase in the percentage of the amorphous portion in PEO/E8/NaIO_4_, thereby facilitating the softening and reducing *T_g_* and *T_m_* compared to the base composite PEO/E8. It should be noted that the amorphous portion undergoes the glass transition only, and the crystalline portion undergoes melting only.

**Figure 3:**
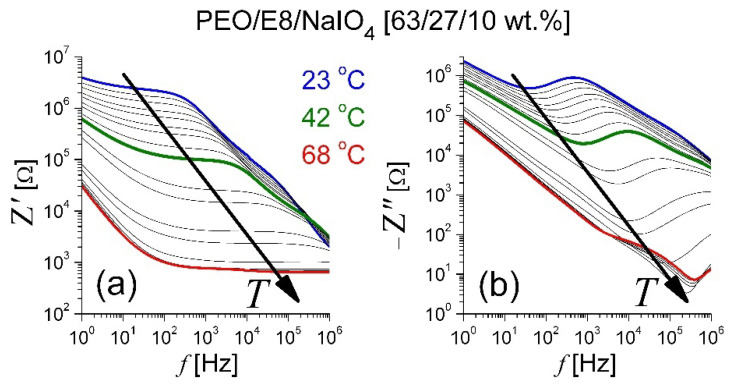
Frequency spectra of complex electrical impedance of PEO/E8/NaIO_4_ composite film: (**a**) real part (*Z′*) and (**b**) imaginary part (*Z″*). Spectra were recorded under identical experimental conditions, by varying the temperature of the film in the range of 22 °C to 68 °C. The arrows show the effect of increasing temperature.

**Figure 4:**
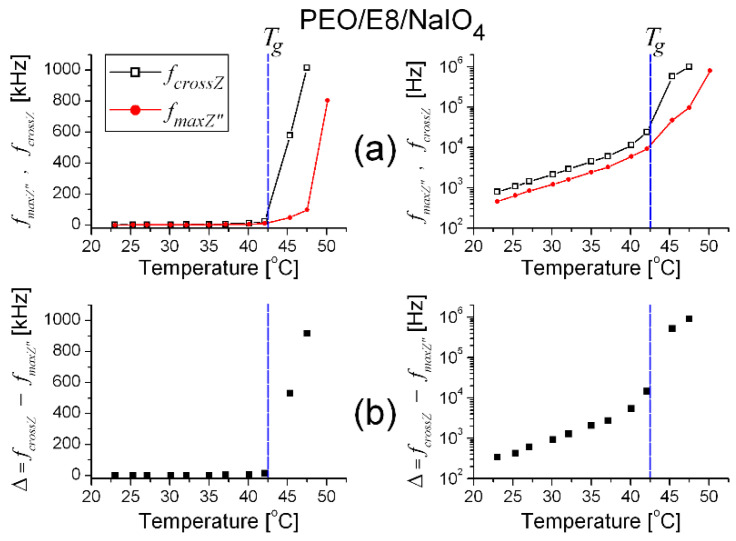
The frequencies *f_cross_* and *f_maxZ″_* (**a**), as well as the difference Δ = *f_cross_* − *f_maxZ″_* (**b**) vs. the temperature for PEO/E8/NaIO_4_ composite, in linear (**left**) and logarithmic (**right**) scale. The dashed lines point out the glass transition (*T_g_*) and melting temperature (*T_m_*) values of the PEO/E8/NaIO_4_ composite. The uncertainty limits for the experimental data presented are within the size of the symbols for the data.

**Figure 5:**
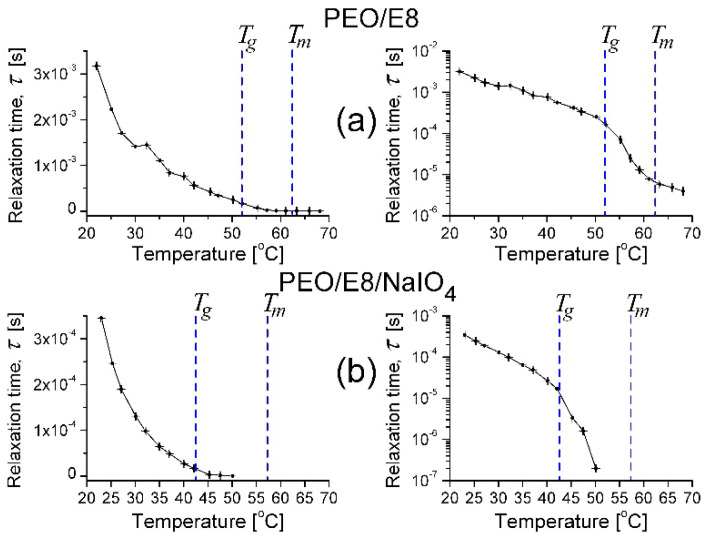
Characteristic dielectric relaxation times τ calculated for PEO/E8 (**a**) and PEO/E8/NaIO_4_ (**b**). The dashed lines point out the values of glass transition temperatures (*T_g_*) and melting temperatures (*T_m_*) for both composites. The error bars represent the uncertainty limits for the measured data. The relative error by evaluation of τ is: *δ*τ/τ = ±*δf/f*, where *δf* = 0.1 Hz is the accuracy of the measured values of the frequency *f*.

By means of analyses of the impedance spectra (Figure 3) of the salt-complexed polymer electrolyte system PEO/E8/NaIO_4_ based on the same PEO:E8LC composition, similar observations took place for the thermo-produced changes (Figure 4). They also suggest dipole reorganization and dielectric relaxation in this ion-conductor polymer-ion coupled composite electrolyte upon AC field. Accordingly, the same conclusions may be drawn, as above. For PEO/E8/NaIO_4_, the observed temperature threshold value *T_th_* was close to the *T_g_* of this composite. Note that the frequencies *f_crossZ_* and *f_maxZ″_* were much higher than those observed by PEO/E8 and became greater than 1 MHz, and therefore, were outside of the working range. A comparison of the corresponding characteristic dielectric relaxation times τ for both polymer-LC composites’ understudies can be seen in Figure 5. The shorter τ for PEO/E8/NaIO_4_ was due to an increase in the segmental motion of polymer chains due to the addition of the salt (see below, in Section 3.2.2).

**Figure 6:**
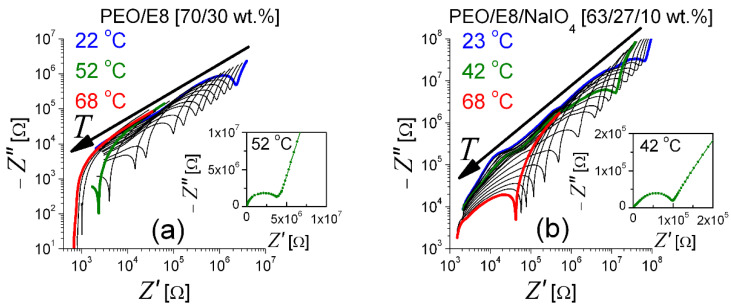
Nyquist plots of PEO/E8 (**a**) and PEO/E8/NaIO_4_ (**b**) at various temperatures. The arrows show the effect of increasing temperature. Inserts: the Nyquist plots that correspond to impedances measured at the glass-transition temperature (*T_g_*) for both composites.

The above results of the thermal behaviors of PEO/E8 and PEO/E8/NaIO_4_ can be highlighted by the presentation of the temperature-dependent {*Z′*, *Z″*} spectra of these composites by means of the corresponding complex impedance diagrams—the Nyquist plots (−*Z″* versus *Z′*). They consisted of semicircle arcs on the high-frequency side, followed by a steep line on their low-frequency side (Figure 6). The semicircle corresponded to bulk material properties (such shapes are relevant to the equivalent circuit of capacitance and resistance connected in parallel). In the lower-frequency region, the gradual increase in absolute *Z″* values as the frequency decreases indicated a build-up of electric double-layer capacitance at the electrode/electrolyte interface. The value of the bulk resistance (*R_B_*) of the material could be determined from the point of intersection of the low-frequency (i.e., high-resistance) end of the depressed semicircle on the *Z′* axis [46,56]. Then, the values of the ion conductivity (σ) of the studied composite films were obtained from *R_B_*.

**Figure 7:**
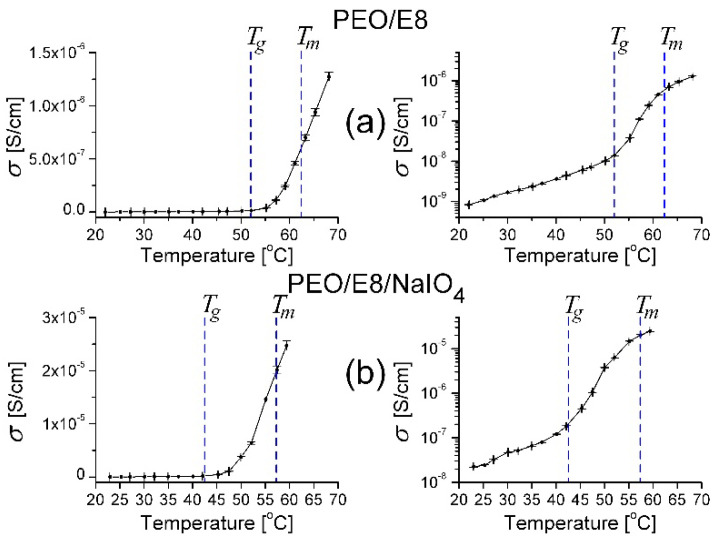
Comparison of the temperature dependencies of ion conductivity (σ) determined for PEO/E8 (**a**) and PEO/E8/NaIO_4_ (**b**). The dashed lines point out the values of glass transition temperatures (*T_g_*) and melting temperatures (*T_m_*) for both composites. The error bars represent the uncertainty limits, discussed in Section 2.

From the temperature dependencies of σ for PEO/E8 (Figure 7a) and PEO/E8/NaIO_4_ (Figure 7b), it is seen that for both composites σ increased with increased temperature. This was reasonable for the studied ion-conducting solid polymer-based systems and has been reported elsewhere after analyses of the corresponding Arrhenius plots [36,38]. Note that the mobile ionic species in PEO/E8 are the H^+^/OH^−^ ions of PEO [57] and ions from “impurities” in E8 LC, the amounts of which are relatively low, while in PEO/E8/NaIO_4_, Na^+^ ions are the most active. As seen from Figure 7, the σ values of these composites abruptly increased above a specific threshold temperature, and these threshold values were very near to the corresponding *T_g_* values for both composites. The temperature behavior of σ and the apparent thresholds of the studied polymer/LC composites were consistent with the increase in segmental motion of the polymer chains at *T* > *T_g_*, according to the dynamic bond percolation theory [58,59,60], the free-volume model of a glassy body and Vogel–Tamman–Fulcher equation [61,62].

### 3.2. Dielectric Spectroscopic Analysis

#### 3.2.1. Dielectric Permittivity

**Figure 8:**
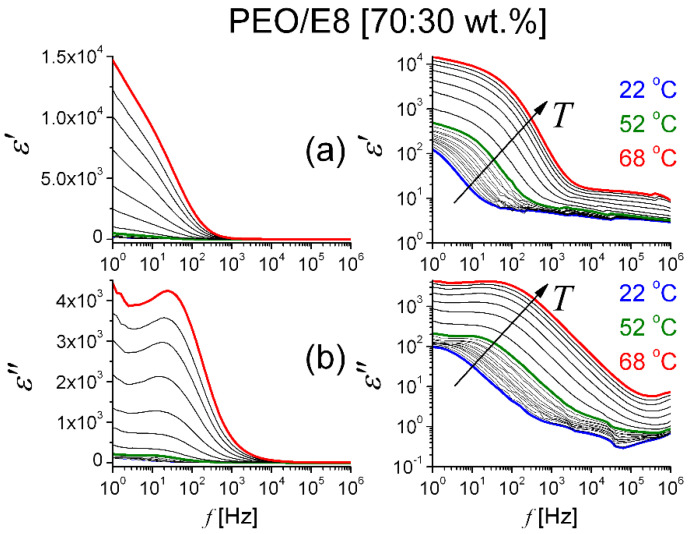
Frequency spectra of real (**a**) and imaginary (**b**) parts of complex dielectric permittivity for PEO/E8 at various temperatures. Data for ε presented in linear (**left**) and logarithmic (**right**) scale. The arrows show the effect of increasing temperature.

**Figure 9:**
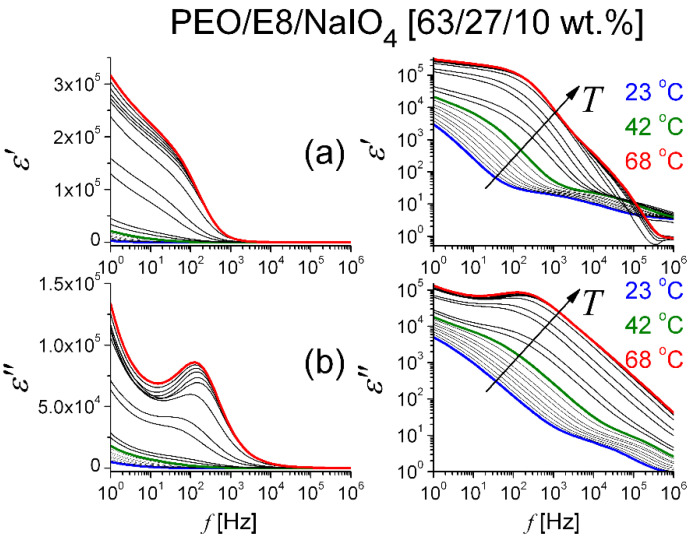
Frequency spectra of real (**a**) and imaginary (**b**) parts of complex dielectric permittivity for PEO/E8/NaIO_4_ at various temperatures. Data for ε presented in linear (**left**) and logarithmic (**right**) scale. The arrows show the effect of increasing temperature.

By means of the expressions

(1) $$\varepsilon^{\prime }\left(f\right)=\frac{-Z^{\prime \prime }\left(f\right)d}{2\pi f{\;\varepsilon }_{0}\;A\;{\left|Z\left(f\right)\right|}^{2}}\;\mathrm{and}\;\varepsilon^{\prime \prime }\left(f\right)=\frac{Z^{\prime }\left(f\right)d}{2\pi f{\;\varepsilon }_{0}\;A\;{\left|Z\left(f\right)\right|}^{2}}$$

from the measured *Z′* (*f*) and *Z″* (*f*) spectra, we calculated the real (*ε′*) and imaginary (*ε″*) parts of the complex dielectric function of the PEO/E8 (Figure 8) and PEO/E8/NaIO_4_ (Figure 9) composites under study. The geometrical parameters were *A* = 1 cm^2^ and *d* = 150 μm, and *ε_o_* = 8.85 × 10^−12^ F.m^–1^ was the value of the permittivity of free space. The dispersion curves for both *ε′* and *ε″* had relaxational characteristics. At a given temperature, the sharp increase in dielectric permittivity at low frequency was related to the electrode polarization [43,45,48], which occurred due to the formation of an electric double layer. In AC fields, the deposed charges forming dipoles at the electrodes led to relaxation behavior that was similar to dipolar relaxation, and the dielectric displayed non-Debye behavior. The frequency behavior of dielectric permittivity of the polymer PEO has been previously investigated in detail, e.g., [63,64]. An essential peculiarity in our case was the triggering of ‘soft mode’ relaxation in the PEO/E8 composite at frequencies that tended to zero (discussed in Section 3.1). The non-linear decrease in both the *ε′* and *ε″* values towards the higher frequency of the applied AC electric field resulted from the contributions of various polarization processes. 

It is possible to observe in Figure 8 and Figure 9 that by increasing temperature *T*, the values of both *ε′* and *ε″* for the two polymer/LC composites were increasing throughout our frequency window. The increases in both *ε′* and *ε″* could be because the increase in temperature favored the orientation of the molecular dipoles upon the applied electric field that could polarize each separate molecule and the dielectric medium as a whole. Further, Figure 8 and Figure 9 show that by gradually increasing the values of *T*, there were obvious temperature thresholds equal to corresponding *T_g_* values of the composites. Below *T_g_*, both *ε′* and *ε″* were slowly increasing functions of *T*, but at *T* > *T_g_*, an enhanced dielectric response was present. Moreover, the heating did result in considerable changes in the spectral behaviors of dielectric permittivity. In particular, the change of dielectric loss (*ε″*) values with *T* signified dipole relaxation and a decrease in the dielectric relaxation time. As a whole, the frequency response of dielectric permittivity of the studied composites agreed with the data reported in the literature for the dielectric response of solid dielectrics and polymer-based solid electrolytes below and above the glass transition temperature [65].

**Figure 10:**
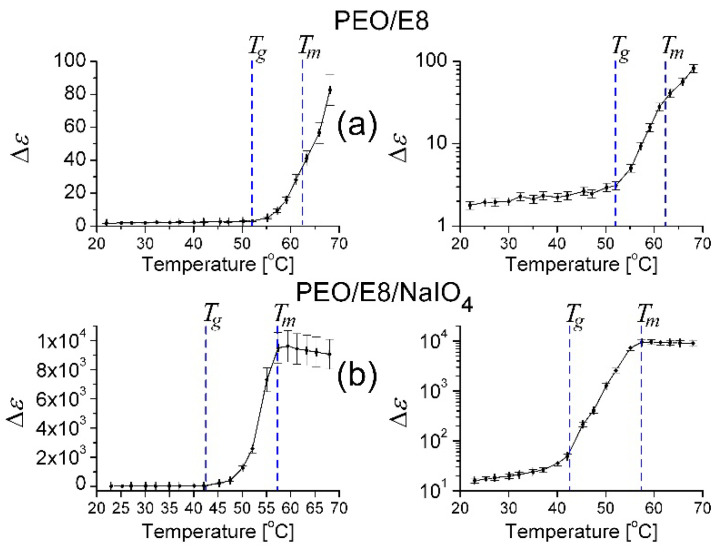
Plots of dielectric relaxation strength Δ*ε* vs. temperature. Data for PEO/E8 (**a**) and PEO/E8/NaIO_4_ (**b**), in linear (**left**) and logarithmic (**right**) scales. The dashed lines point out the values of glass transition temperatures (*T_g_*) and melting temperatures (*T_m_*) for both composites. The error bars indicate the uncertainty of the calculated Δ*ε* values discussed in the text.

From *ε′* dielectric spectral data, one can estimate the dielectric relaxation strength (the dielectric strength of a relaxation process; the dielectric intensity) Δ*ε* = *ε′*_s_ − *ε′*_∞_ [43,45,46,66], where *ε′*_s_ and *ε′*_∞_ are the values of *ε′* corresponding to the static dielectric permittivity (at relatively low frequency) and high-frequency limited dielectric permittivity of the film, respectively. The quantity Δ*ε* is a measure of the dipole polarization. It can be seen from the *ε′*(*f*) spectra that at *f* ≥ 1 kHz, the contribution of the electrode polarization process was largely suppressed, and the bulk material properties started to dominate in the contribution of dielectric polarization. The leveling-off at higher frequencies represents the atomic and electronic polarization along with some contribution of dipolar polarization in the PEO/E8 film. As a reasonable approximation, one can take Δ*ε* = *ε′*(1 kHz) − *ε′*(1 MHz). Figure 10 presents the temperature dependence of Δ*ε* for PEO/E8 (Figure 10a) and PEO/E8/NaIO_4_ (Figure 10b). As with the σ values, significant error contributors to the calculated values of Δ*ε* were the uncertainties of both the measured thickness *d* of the films and the electrode sizes (*s*). With the relative error of 1% (maximum) for the measured impedance *Z*, the overall uncertainty for the calculated *ε* values was ±5.6%, and correspondingly, it was ±11.2% for the values of Δ*ε*.

As seen in Figure 10, Δ*ε* gently increased as *T* increased up to *T_g_*, and was strongly enhanced with elevating *T* in a narrow temperature range between *T_g_* and *T_m_*. In the vicinity of *T_m_*, Δ*ε* continued to increase with the increase in *T*, but at *T_m_*, the slope of the Δ*ε*(*T*) curves was reduced, in agreement with results reported for PEO [55,63], and even a decrease in Δ*ε* was observed for PEO/E8/NaIO_4_. The effect of melting on the dielectric behavior of semi-crystalline, linear, polar polymers, such as PEO, is well established—the relaxation strength in the melt is much greater than that of the solid, and the relaxation strength of the partially melted material is somewhere between that of the solid and that of the melt [55,63].

In our case, the dielectric increment with temperature suggested a dipole contribution to the polarization. As such, the Δ*ε*(*T*) behaviors between *T_g_* and *T_m_* implied an enhanced electro-dipolar character and an increase in polarization in the considered polymer/LC composites. The temperature dependence of Δ*ε* showed the thermo-produced change in their dipolar nature (and dipole–dipole interaction). The enhancement of Δ*ε* by softening was relevant to increasing the free space between and along the polymer chains and, thereby, to the enhanced orientational motion of the molecular dipoles. In relation to this, the above results for Δ*ε*(*T*) confirm that by elevating *T*, the localized dipolar motion/orientation became less restricted by surrounding polymer chains, especially above *T_g_*.

The comparison of Δ*ε* calculated for PEO/E8 composite and the polymer PEO (using impedance data shown in Figure S4) at room temperature showed nearly the same values (Δ*ε*~2). In contrast, a much higher value (Δ*ε*~20) was calculated for pure E8LC (using the impedance spectra shown in Figure S4). Thus, there was practically no increase in Δ*ε* of PEO/E8, contrary to the expectation that Δ*ε* would be increased by including highly polar molecules of E8 LC at a relatively high concentration of 30 wt% in the polymer PEO. This fact suggests a coupling of most of the dipoles of E8LC to the PEO polymer chain structure, thus neutralizing their dielectric strength.

Note that the values of Δ*ε* for PEO/E8/NaIO_4_ at a given value of *T* (Figure 10b) were much higher than Δ*ε* for PEO/E8 (Figure 10a). The reason for this cannot be only the increase in free volume in the amorphous region of PEO/E8 composite through the addition of the salt NaIO_4_ (and the formation of polymer–salt complexes). Ultimately, the same effect resulted in easier transport of mobile ion species, and also to enhanced mobility of ions supported by an increased segmental motion of the polymer chains, leading to the enhanced ion conductivity of this electrolyte system, as mentioned in Section 3.1. The significant difference in Δ*ε* (i.e., the difference in the density of dipole moments) for the two composites implied different amounts and/or different strengths of electrically active molecular dipoles, or/and different kinds of molecular dipoles in these two composites.

#### 3.2.2. Dielectric Loss Tangent and AC Conductivity

**Figure 11:**
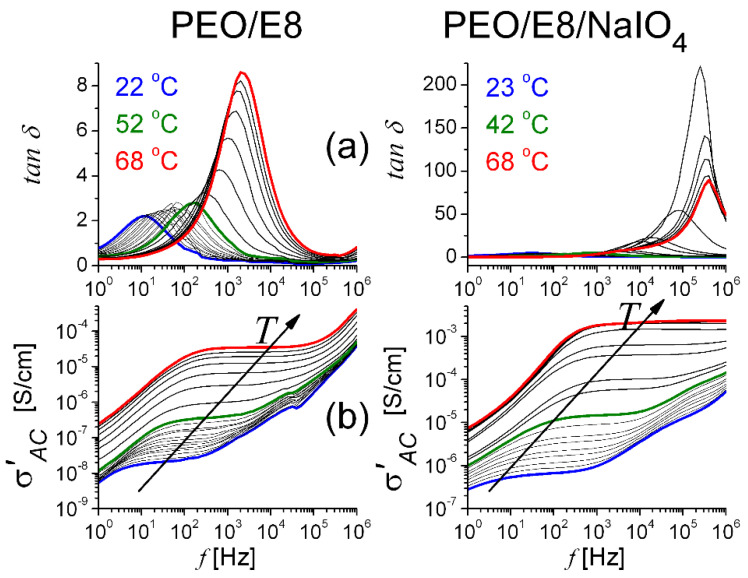
The variation of frequency-dependent dielectric loss tangent *tanδ* (**a**) and the real part of AC conductivity $\sigma_{AC}^{’}$ (**b**) at various temperatures for the studied polymer/LC composites PEO/E8 (**left**) and PEO/E8/NaIO_4_ (**right**). The arrows show the effect of increasing temperature.

In general, the dielectric losses, as relaxation phenomena, are due to dipole losses (dissociation of the dipolar formation), conductivity losses, and vibration losses [43,45,48,55,64,67]. Conduction losses, being proportional to (*σ*/*ω*) (where, σ is the electrical conductivity, and ω is the angular frequency), rise with increasing temperature, which in turn causes an increase in the value of dielectric losses. From the calculated *ε*(*f*) spectra (Figure 8 and Figure 9), we obtained the dielectric loss tangent *tanδ*(*f*) = ε″(*f*)/ε′(*f*) and the real part of the AC conductivity $\sigma_{AC}^{’}\left(f\right)=$2π*f* *ε_o_* *ε″*(*f*) [68,69]—characteristics that can also give valuable information about the dipole dielectric relaxations and the corresponding relaxation mechanisms in the dielectric materials. As known, *tanδ* is indicative of the polymer chain relaxation behavior. The peak of the *tanδ* spectrum corresponds to the polarization relaxation frequency. This frequency was near the characteristic frequency *f_min_* in the *Z″*(*f*) spectrum (Figure S5). The $\sigma_{AC}^{’}\left(f\right)\;$ characteristics represent the dissipation of energy. Figure 11 shows the calculated *tanδ*(*f*) and $\;\sigma_{AC}^{’}\left(f\right)$ for the studied PEO/E8 and PEO/E8/NaIO_4_ composites, in terms of their dependence on temperature. As for *ε*(*f*) spectra, with the increasing of the temperature, a clear enhancement for *tanδ*(*f*) and $\;\sigma_{AC}^{’}\left(f\right)$ was observed above a threshold equal to the temperature that corresponded to the *T_g_* value for each of these composites. The most significant features of these behaviors were the apparent shift of the *tanδ* peak towards higher frequencies and the increase in $\sigma_{AC}^{’}\;$ values on heating.

Over the whole range measured from 1 Hz to 1 MHz, the temperature-dependent variations of the $\sigma_{AC}^{’}$ values followed the same trend as the ionic conductivity σ discussed in Section 3.1, i.e., for each of the studied composites, the dependence of $\sigma_{AC}^{’}$ vs. temperature at a given frequency value *f* would display a dependence similar to the corresponding one seen in Figure 7. Regarding the variation in $\sigma_{AC}^{’}\left(f\right)$ at various temperatures, three distinct regions were observed in the plots for both PEO/E8 and PEO/E8/NaIO_4_ composites: (i) a low-frequency region due to electrode polarization; (ii) a mid-frequency region where $\sigma_{AC}^{’}\;$ was independent (or weakly dependent) on *f* (this flat region was a result of the diffusion of ions when *f* was lower than the hopping frequency); and (iii) a high-frequency region, in which *f* exceeded the hopping frequency. The crossover from frequency-independent conductivity to dispersion depicted the relaxation phenomenon. These plots were typical for dry-state polymer-based ion electrolytes.

**Figure 12:**
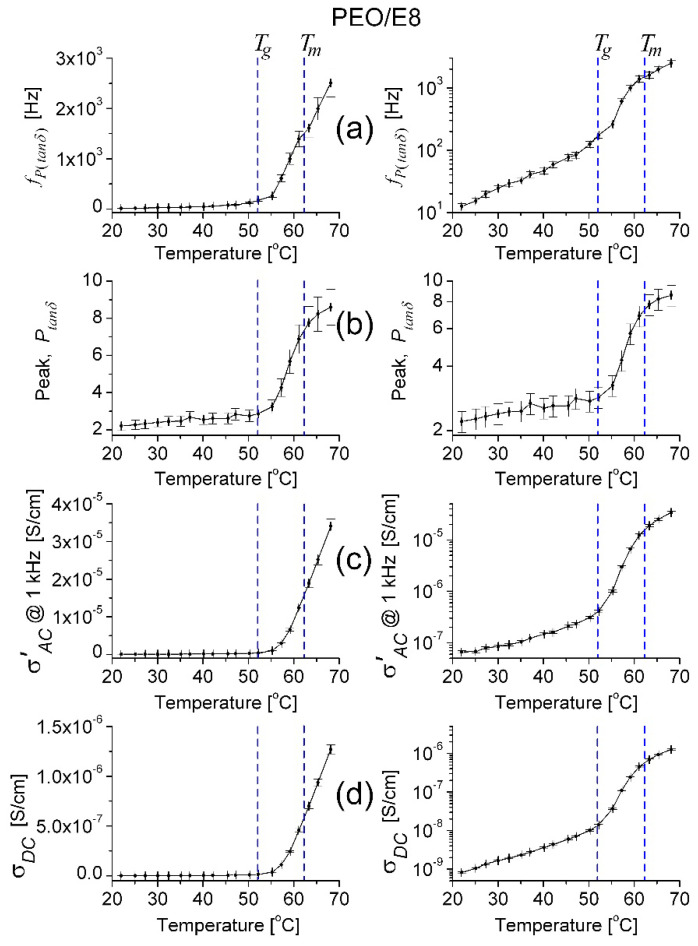
Temperature dependence of the peak frequency *f_P_*_(*tanδ*)_ (**a**)**,** and the peak values *P_tanδ_* (**b**) and $\sigma_{AC}^{’}\;$ at *f* = 1 kHz (**c**)**,** determined for PEO/E8 composite, in linear (**left**) and logarithmic (**right**) scale. The temperature dependence of ion conductivity (σ) for PEO/E8 is given for comparison (**d**). The dashed lines point out the values of glass transition temperatures (*T_g_*) for both composites. The error bars indicate the uncertainty of the calculated values.

Regarding the temperature dependencies of the values of frequency *f_P_*_(*tanδ*)_ and the maximum (*P_tanδ_*) of the *tanδ* peak (Figure 12a,b, respectively) by PEO/E8, similarly to the temperature behaviors for *f_maxZ”_* and *f_crossZ_* obtained from impedance spectra (recall Figure 5), they exhibited temperature thresholds close to the one of the ionic conductivity $\sigma_{AC}^{’}\left(T\right)$ for PEO/E8 (Figure 12d), i.e., a threshold equal to *T_g_*(PEO/E8). The same applied to the temperature behavior $\sigma_{AC}^{’}\left(T\right)$ for PEO/E8 at a given frequency, e.g., *f* = 1 kHz (Figure 12c). The observed temperature behaviors were attributed to the increased free volume in the amorphous region of the PEO/E8 composite at *T* > *T_g_*, as explained in Section 3.1. Through the melting point, *f_P_*_(*tanδ*)_ is a continuous function with a smaller increment, as measured for PEO [55,63].

**Figure 13:**
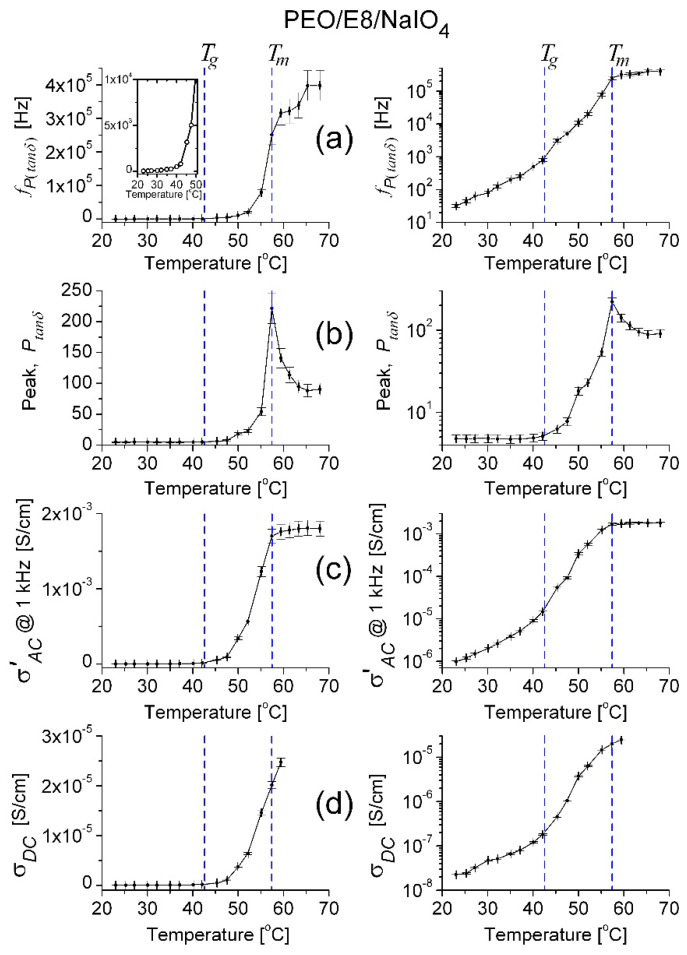
Temperature dependence of the peak frequency *f_P_*_(*tanδ*)_ with the insert representing the enlarged part of the temperature dependence *f_P_*_(*tanδ*)_(*T*) (**a**)**,** and the peak values *P_tanδ_* (**b**) and $\sigma_{AC}^{’}\;$(**c**) at *f* = 1 kHz determined for PEO/E8/NaIO_4_ composite, in linear (**left**) and logarithmic (**right**) scale. The temperature dependence of ion conductivity (σ) for PEO/E8 is given for comparison (**d**). The dashed lines point out the values of glass transition temperatures (*T_g_*) for both composites. The error bars indicate the uncertainty of the calculated values.

With PEO/E8/NaIO_4_, the temperature dependence of *f_P_*_(*tanδ*)_ also exhibited a distinct temperature threshold at *T_g_*(PEO/E8/NaIO_4_) = 42.5 °C (the insert in Figure 13a); however, it was somewhat smoothed and extended at the temperature scale (Figure 13b). Thus, the threshold was integrally observed to be at ca. 50 °C (Figure 13a), i.e., considerably higher than *T_g_*(PEO/E8/NaIO_4_) = 42.5 °C. Indeed, the observed threshold temperature was nearer to *T_g_*(PEO/E8) than to *T_g_*(PEO/E8/NaIO_4_). Thus, there was a significant difference when comparing *f_P_*_(*tanδ*)_(*T*) to the behaviors represented by $\sigma_{AC}^{’}\left(T\right)$ (Figure 13c) and $\sigma_{DC}\left(T\right)$ (Figure 13d) obtained for the same PEO/E8/NaIO_4_ sample. Since the threshold temperatures observed in the $\sigma_{AC}^{’}\left(T\right)$ and $\sigma_{DC}\left(T\right)$ dependencies were related to the thermally dependent segmental motion, these temperature values were close to *T_g_*(PEO/E8/NaIO_4_). The observation of the smoothed/extended threshold, as shown in Figure 13a, reflected the fact that the ionic compound NaIO_4_ and Na^+^ ions did influence the polymer chain motion, as well as the dipole–dipole interaction, in the composite PEO/E8/NaIO_4_.

The thermo-induced change of the dielectric loss factor of PEO/E8/NaIO_4_ was influenced by Na^+^ ions in the structure of this salt-complexed polymer/LC composite (metal ion-coupled polymer/LC electrolyte) with the presently used NaIO_4_ salt content of 10 wt.%. The addition of the ionic inorganic compound NaIO_4_ did considerably change the structure of the PEO/E8 composite and the molecular dipole formation. The Na^+^ ions from NaIO_4_ tended to form links with ether oxygen atoms in the flexible ethylene oxide segments in PEO/E8/NaIO_4_ [38], thereby Na^+^ ions and NaIO_4_ did compete for the intermolecular coupling between PEO and E8LC in the same chemical structure. Thus, the possible coupled molecular organic dipolar formations PEO-E8LC (Figure S1b) in PEO/E8/NaIO_4_ were much less than in PEO/E8 composite.

**Figure 14:**
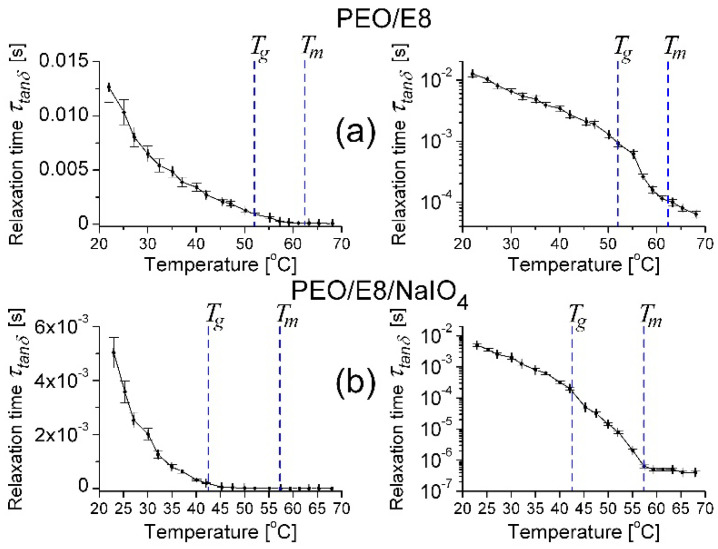
Temperature dependencies of the relaxation times corresponding to the frequency *f_P_*_(*tanδ*)_ for PEO/E8 (**a**) and PEO/E8/NaIO_4_ (**b**) composites, in linear (**left**) and logarithmic (**right**) scale. The dashed lines point out the values of glass transition temperatures (*T_g_*) for both composite systems. The error bars represent the uncertainty for the calculated values.

The Na^+^ ions brought a large ionic conductivity (that was reflected in ion conductivity values of the PEO/E8/NaIO_4_ electrolyte system being nearly one order of magnitude higher as compared to the base PEO/E8 (recall Figure 7)).In addition, the sizable improvement of the amorphous phase owing to the salt NaIO_4_ [38] contributed to the alteration of the dielectric properties, dielectric relaxation, and to a reduction in the characteristic dielectric relaxation time for PEO/E8/NaIO_4_ (recall Figure 5), seen also by thermo-induced changes in the polymer chain relaxation time τ*_tanδ_* = (2π *f_P_*_(*tanδ*)_)^–1^ for both PEO/E8 and PEO/E8/NaIO_4_ (Figure 14). In both cases, the decrease in the relaxation time with the increasing of the temperature was due to the faster movement of Na^+^ ions in the AC field.

Most important was the large thermo-induced shift of the position of the peak *tanδ* towards the higher frequency region. This shift, seen by PEO/E8/NaIO_4_, was 1–2 orders of magnitude higher concerning PEO/E8 (Figure 12a). Actually, in Figure 13a, the temperature dependence of the peak frequency *f_P_*_(*tanδ*)_ is present, and the temperature dependence of the thermo-induced shift is the same. In general, such shifts are attributed to a decrease in the degree of crystallinity with a corresponding increase in the amorphicity of the polymer matrix, thereby enhancing the flexibility and increasing the segmental motion of polymer chains controlling the ionic and AC conductivity [45]. The higher the shift of *f_P_*_(*tanδ*)_, the higher the segmental motion of the polymer chains. The structural reorganization of PEO upon the addition of 10 wt.% NaIO_4_ salt and the complete dissociation of the salt NaIO_4_ in the polymer matrix of the PEO/E8/NaIO_4_ electrolyte system at a molecular level, resulting in a successive polymer–salt complexation, were evidenced by XRD and Raman studies [38].

#### 3.2.3. Modelling of Dielectric Behaviors

To further characterize the studied PEO/E8 and the salt-complexed Na^+^ ion conductor PEO/E8/NaIO_4_ ion-polymer-LC composite, we modelled the frequency spectra of their dielectric permittivity and their thermo-induced changes. Firstly, we tried to fit the *ε″*(*f*) spectra in the frame of the common conventional models. However, fits using either the Havriliak–Negami (HN) relaxation function [70,71] or the Kohlrausch–Williams–Watts (KWW) function [72,73] (using a single or two related relaxation terms, corresponding to each relaxation mode), and with the standard addition of conductivity contributions that were significant at lower frequencies, were unsuccessful. As known, the HN relaxation function is relevant for the modelling of the relaxation behavior of LCs and LCs-based media [45,74]. The KWW model has been successfully applied via the analysis of dielectric relaxations in solid-state polymeric materials [75,76,77,78,79,80,81,82], non-polymeric and polymeric glass-forming systems [83], as well as liquid crystalline side-chain polymers (a flexible-chain backbone to which mesogenic groups are attached either longitudinally or transversely via a spacer group, which is typically a CH_2_, or other variants [76]).

In particular, in our case, one can adequately fit the spectral data of the complex permittivity *ε″* (the dielectric loss) with a combination of both the KWW and HN functions. Such an approach is a generalization of Debye relaxation model, and the contributions of both components of the PEO/E8 composite (the polymer and the LC) in the dielectric behavior can be considered [38,84]. Thus, we used the following function to fit the *ε″*(*f*) spectra:

(2) $$\varepsilon^{\prime \prime }\left(f\right)=\frac{\sigma_{dc}}{\varepsilon_{0}{\left(2\pi f\right)}^{n}}+F.T.\;\left\{\Delta \varepsilon_{KWW}\;\exp\left[-{\left(\frac{t}{\tau_{KWW}}\right)}^{\beta }\right]\right\}+\frac{\Delta \varepsilon_{HN}}{{\left[1+{\left(f/f_{0}\right)}^{a}\right]}^{b}}$$

where the first term is the conductivity term, the second is the time-domain relaxation KWW term (stretched exponent of time), and the third—the HN term, $\sigma_{dc}$ (S/cm)—is the asymptotic conductivity at the 0 Hz limit (the so-called DC electrical conductivity); *f* (Hz) is the frequency of the applied electric field, *f*_0_ (Hz) is the relaxation frequency, ε_0_ is the vacuum permittivity, Δ*ε* is the dielectric strength, *n* is the power factor of the conductivity term. The empirical parameters *a* and *b* (∈[0, 1]) refer to the shape of the relaxation spectra accounting for the asymmetry and broadness of the dielectric dispersion curve (power indexes that define the low- and high-frequency limits of *ε″*, respectively). *F*.*T*. means the Fourier transform, *β* is the stretching exponent, $\tau_{KWW}\;$(s) is dielectric relaxation time, and *t* is the time variable. 

**Figure 15:**
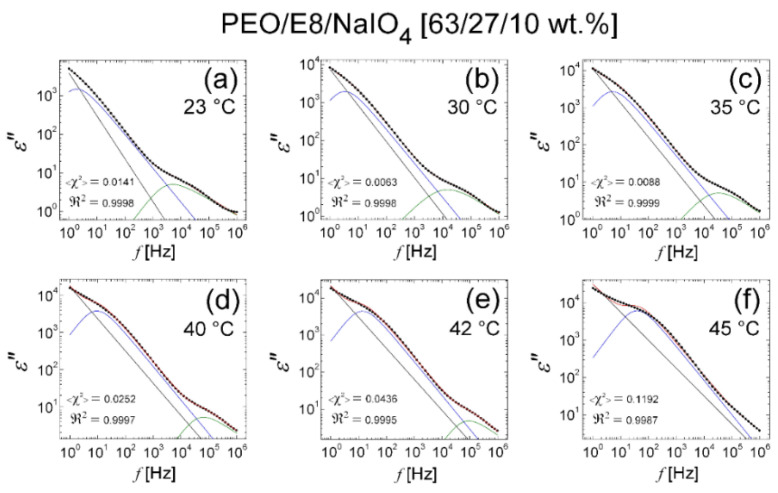
Dielectric spectra *ε″*(*f*) of PEO/E8/NaIO_4_ composite at various temperatures in the range of 23 °C (*T_room_*) to 45 °C (slightly above *T_g_* = 42.5 °C): 23 °C (**a**); 30 °C (**b**); 35 °C (**c**); 40 °C (**d**); 42 °C (**e**); 45 °C (**f**). The *ε″* spectral data are represented by the symbol (X); the lines are the best fits to the *ε″* data with the use of fitting function in Equation (2) (KWW + HN model). The curves represent the two resolved (decomposed) peaks of the dielectric relaxation response in the *ε*″(*f*) spectrum, resulting from the KWW function (blue) and the HN function (green), respectively; the straight line represents the contribution of the conductivity term; the envelope of the three spectral components’ curves (the total fit) is colored in red. As quality measures of each obtained fit, the values of the reduced <*χ*^2^**>** factor (the coefficient of determination by fit), as well as the regression coefficient $\left({ℜ}^{2}\right)$ (the goodness of fit), are also shown.

**Figure 16:**
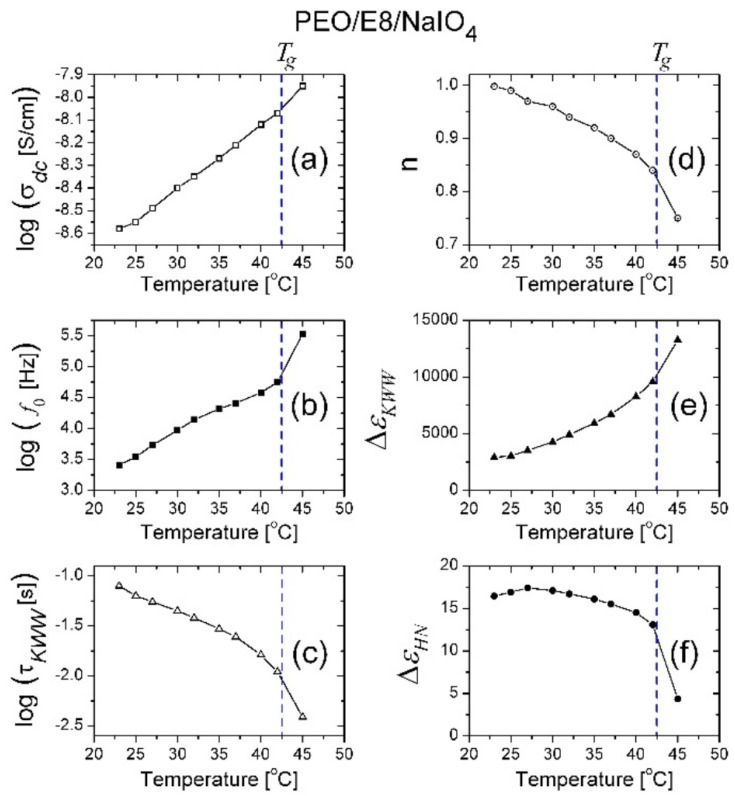
Temperature dependencies of the values of parameters derived by the best fits using Equation (2) (KWW + HN model): $\sigma_{dc}$ (**a**); *f*_0_ (**b**); $\tau_{KWW}$ (**c**); *n* (**d**); $\Delta \varepsilon_{KWW}$ (**e**); $\Delta \varepsilon_{HN}$ (**f**). The fits were performed on the *ε*″(*f*) spectra of the PEO/E8/NaIO_4_ composite (the fits in Figure 15) that correspond to temperatures from 23 °C to 45 °C.

The results from the fits on the *ε″* dielectric spectral data for the PEO/E8/NaIO_4_ composite performed using Equation (2) are shown in Figure 15. It was found that at temperatures from *T_room_* up to *T* = 40 °C, the fits were perfectly satisfying, and to some extent, approximately acceptable at *T* = 42 °C (Figure 15e). Significantly, in temperature dependencies of the values of the derived best-fit parameters (Figure 16), there was a gradual increase in $\sigma_{DC}$, $f_{0}$ and $\Delta \varepsilon_{KWW}\;$ with the increasing of *T*, together with a steep increasing trend at *T* ≥ *T_g_*(PEO/E8/NaIO_4_) = 42.5 °C, as well as a gradual decrease in *n*, τ and $\tau_{HN}$ together, with a steep decrease in their values at *T* ≥ 42.5 °C. These observations agree with the temperature threshold found by analyzing the of complex impedance spectra of the considered PEO/E8/NaIO_4_ composite and demonstrated in the results discussed in Section 3.1. In particular, the reduction in the values of the power factor *n* with the increasing temperature (Figure 16d) indicated a deviation from Debye’s model, thus confirming the conclusions in Section 3.1. This could be interpreted as a deviation from the ideal dielectric material, for which *n* = 1. Such a change could also be associated with the thermo-produced increase in the inhomogeneity of the dielectric PEO/E8/NaIO_4_. Note that the reduction in *n* by heating the studied polymer-LC composite system could be straightforwardly related to the change in the dipolar organization. The latter should strongly affect the dipolar polarization, as well as the electrical conductivity in the system.

**Figure 17:**
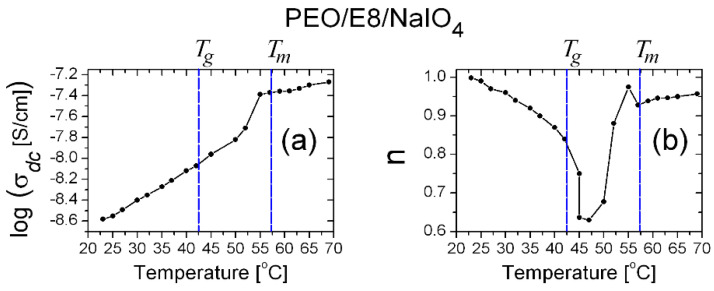
Temperature dependencies of the parameters σ*_dc_* (**a**) and *n* (**b**) obtained by fits using Equation (2) (KWW + HN model). The fits were performed on *ε″*(*f*) spectra of the PEO/E8/NaIO_4_ composite film that corresponded to temperature in the range of 23–68 °C.

At T ≥ 45 °C it was possible to obtain reasonable fits on the *ε″*(*f*) spectra of the PEO/E8/NaIO_4_ composite with Equation (2) that gave meaningful values only for the conductivity term (Figure 17). It is well known that in dipolar materials undergoing the glass transition, such as glasses and many polymers, there are fundamental differences between the responses below and above *T_g_*. There are specific processes below *T_g_* where the composite is rigid, and other processes above *T_g_* where the composite may be considered soft (or liquid-like), due to the coexistence of crystalline and amorphous phases of PEO below *T_m_*. Though the dielectric response of the considered ion-conductive material became more complex in this case, the change in these parameters at a temperature close to *T_g_* was evident. Our analyses indicated that at *T* > *T_g_*, the two main spectral components—peaks (and their convolutions)—were considerably changed. The peaks corresponded to the two dielectric materials in the composite characterized by the KWW model (for the polymer) and the HN model (for the LC), respectively.

In general, the dielectric data for mixed polymer-LC composites, e.g., polymer-dispersed or disposed liquid crystals (PDLCs) and LCs embedded (LCs-filled) in polymer structures (phase-separated, or bonded), can provide information about the electrical conduction, dielectric relaxation, and reorientation motions of dipoles formed in these soft-solid composite media, due to the fact that they are strongly heterogeneous systems. At the polymer-to-LC composition ratio of 70:30 wt.%, the dielectric behavior and dipole relaxation processes in such composites can be expected to be intermediate between the polymer and the LCs [85,86,87,88]. The dielectric relaxation in polymer-LC composites is a rather complicated process to which various causes likely contribute, e.g., the dipole motions in the LC phase and the dipole motions in the host polymer matrix [76,89]. Even the slightly polar semi-crystalline polymer PEO itself exhibits multiple dielectric relaxations in the amorphous region; this is due to micro-Brownian motions of chains and due to the partial reorientation of chain segment dipoles [75,90,91]. In particular, the considered PEO/E8 and PEO/E8/NaIO_4_ polymer-LC composites are extremely complex heterogeneous systems with complex geometries and morphologies, comprising LC nematic molecules confined in an amorphous (semi-crystalline) polymeric matrix (LC nematic molecules linked to molecular chains in a porous polymer network). Moreover, the LC component E8 in these composites is a eutectic nematic LC mixture of several reactive cyanophenilic/cyanoterphenyl polar compounds. For this reason, to obtain optimal descriptions and adequate modelling of the frequency spectra of the dielectric permittivity of the studied polymer/LC composites, as well as their thermo-induced changes in the *T_g_*-region, it was necessary to apply a more suitable physical model of dielectric behavior that accounted for the thermo-induced changes of the studied composite molecular systems. One example of such a model is the HN model modified with the derivative analytic approach proposed by Wübbenhorst et al. [92]. With this model, the following function was used to fit the dielectric spectra *ε″*(*f*):

(3) $$\varepsilon^{\prime \prime }\left(f\right)=\frac{\sigma }{\varepsilon_{0}{\left(2\pi f\right)}^{n}}+\varepsilon_{\mathrm{der}}^{\prime \prime }$$

where

(4) $$\varepsilon_{der}^{\prime \prime }=\Sigma_{j=1}^{2}\left\{-\frac{\pi }{2}\frac{m_{j}n_{j}\Delta \varepsilon_{j}{\left(\frac{f}{f_{0}{}_{j}}\right)}^{m_{j}}\cos\left[\frac{m_{j}\pi }{2}-\left(1+n_{j}\right)\theta_{\mathrm{HN}}{}_{j}\right]}{{\left[1+2{\left(\frac{f}{f_{0}{}_{j}}\right)}^{m_{j}}\cos\left(\frac{m_{j}\pi }{2}\right)+{\left(\frac{f}{f_{0}{}_{j}}\right)}^{2m_{j}}\right]}^{\left(1+n_{j}\right)/2}}\right\}$$

and

(5) $$\theta_{HN}=\arctan\left\{\sin\left(\frac{m\pi }{2}\right)/\left[{\left(\frac{f}{f_{0}}\right)}^{-m}+\cos\left(\frac{m\pi }{2}\right)\right]\right\}$$

For the values of the shape parameters *m* and *n*, the limit of 0 < *m*,*n* ≤ 1 (*n* might exceed 1, but the product *mn* must be *mn* ≤ 1) was applied.

**Figure 18:**
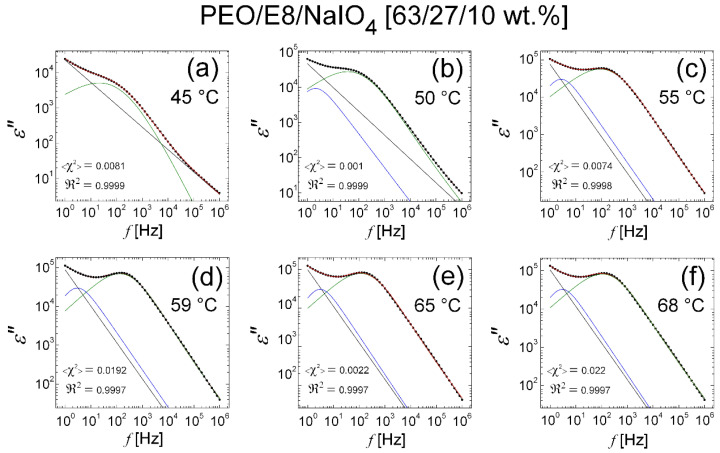
Fits on the *ε″*(*f*) spectra of PEO/E8/NaIO_4_ using Equation (3). The *ε″*(*f*) spectra correspond to temperature range of 45 °C to 68 °C: 45 °C (**a**); 50 °C (**b**); 55 °C (**c**); 59 °C (**d**); 65 °C (**e**); 68 °C (**f**). The *ε″* spectral data are represented by the symbol (X); the blue and the green lines of the best fits on the *ε*″ data are the contributions of the two HN functions in the $\varepsilon_{der}^{\prime \prime }$ term; the straight line represents the contribution of the conductivity term; the envelope of the three spectral components’ curves (the total fit) is colored in red. The values of the reduced <*χ*^2^> factor (the coefficient of determination of fit), as well as the regression coefficient $\left({ℜ}^{2}\right)$ (the goodness of fit), are also shown.

**Figure 19:**
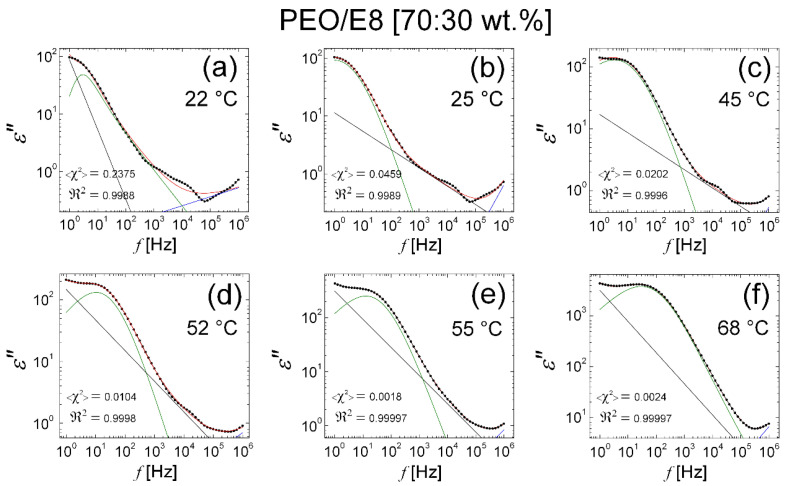
Fits on *ε″*(*f*) spectra for PEO/E8 using Equation (3). The *ε″*(*f*) spectra correspond to temperature range of 22 °C to 68 °C: 22 °C (**a**); 25 °C (**b**); 45 °C (**c**); 52 °C (**d**); 55 °C (**e**); 68 °C (**f**). The *ε*″ spectral data are represented by the symbol (X); the blue and the green lines of the best fits on the *ε″* data are the contributions of the two HN functions in the $\varepsilon_{der}^{\prime \prime }$ term; the straight line represents the contribution of the conductivity term; the envelope of the three spectral components’ curves (the total fit) is colored in red. The values of the reduced <*χ*^2^> factor (the coefficient of determination of fit), as well as the regression coefficient $\left({ℜ}^{2}\right)$ (the goodness of fit), are also shown.

As seen from Figure 18 and Figure 19, the fits with Equation (3) on the *ε″*(*f*) spectra of both the PEO/E8/NaIO_4_ and PEO/E8 composites considered here were satisfying for the *ε″* data corresponding to the polymer softening at *T* ≥ *T_g_*. Because of the presence of a small peak at ~10 KHz, the fits on the *ε″*(*f*) spectra of PEO/E8 at temperatures from *T_room_* to *T_g_* gave relatively poor results (Figure 19a–c). This peak was due to the polarization relaxation of PEO [93] (Figure S6). It should be noted that functions of the KWW form, as well as the KWW + HN model (Equation (2)), were not suitable to appropriately fit the *ε*″(*f*) spectra of PEO/E8 in this temperature range. Due to the strong coupling between ionic and polymer segmental motions, the dielectric response of this polymer-LC composite was not a superposition of KWW + HN responses, even in the temperature range from *T_room_* to *T_g_*. Such a contrast to the case observed with the PEO/E8/NaIO_4_ composite indicated that the dielectric behavior of the studied PEO/E8 composite could not be regarded as a convolution of individual contributions of LC dipoles, nor the polar molecular structure inherent for the pure polymer PEO. Hence, with the PEO/E8LC composite, there may have been dipolar molecular formations arising from the intermolecular coupling between PEO and E8LC (Figure S1b). The fact that, in a certain temperature range (from *T_room_* up to softening at *T_g_*), the combination of KWW + HN formalisms was capable of describing the dielectric behavior of PEO/E8/NaIO_4_ can be explained by the competition between the NaIO_4_–PEO interaction and the PEO-E8LC intermolecular coupling, mentioned in Section 3.2.2. Moreover, the presence of the salt NaIO_4_ led to polarization relaxation and dielectric response, in which the polarization relaxation in PEO was less accentuated (and even suppressed). This effect was well established by the studies of the dielectric permittivity of salt-complexed solid polymer electrolytes, e.g., PEO-LiClO_4_ [94]. Indeed, the relaxation peak at about 10 kHz in the *ε″*(*f*) spectra of PEO/E8 at temperatures of less than *T_g_* (Figure 19a–d) was not observable in the *ε″*(*f*) spectra for the PEO/E8/NaIO_4_ composite (Figure 18).

**Figure 20:**
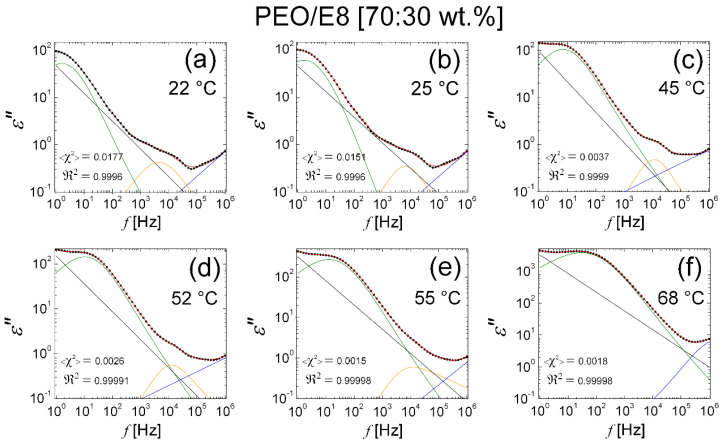
Fits on the *ε″*(*f*) spectra of PEO/E8 using Equation (6). The *ε″*(*f*) spectra correspond to temperature 22 °C (**a**); 25 °C (**b**); 45 °C (**c**); 52 °C (**d**); 55 °C (**e**); 68 °C (**f**). The *ε*″ spectral data are represented by the symbol (X); the blue, the green, and the yellow lines of the best fits on the *ε″* data are the contributions of the two HN functions in the $\varepsilon_{der}^{\prime \prime }$ term and the single HN term in Equation (6), respectively; the straight line represents the contribution of the conductivity term; the envelope of the four spectral components’ curves (the total fit) is colored in red. The values of the reduced <*χ*^2^> factor and the regression coefficient $\left({ℜ}^{2}\right)$ are also shown.

**Figure 21:**
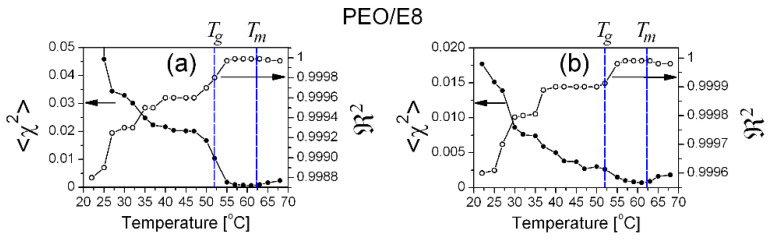
The values of the reduced <*χ*^2^**>** factor (the coefficient of determination of fit) and the regression coefficient $\left({ℜ}^{2}\right)$ (the goodness of fit) against the temperature, for fits of the *ε″*(*f*) spectra of PEO/E8 obtained using: (**a**) Equation (3); (**b**) Equation (6).

**Figure 22:**
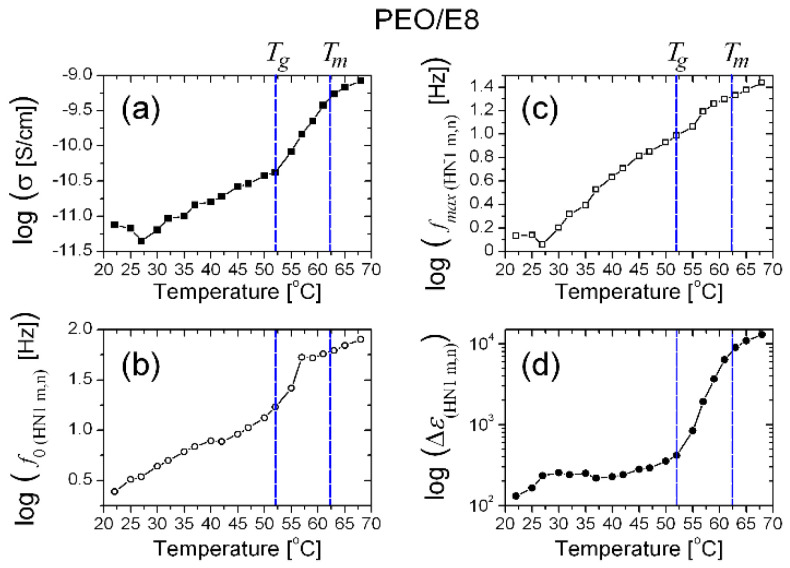
Temperature dependencies of the parameters σ (**a**), *f_0_* (**b**), *f_max_* (**c**) and Δ*ε* (**d**) obtained by fits on the *ε″*(*f*) spectra of the PEO/E8 composite (*ε″*(*f*) spectra in Figure 20) using Equation (6).

Finally, it must be noted that the excellent fits on the *ε″*(*f*) spectra of both the PEO/E8/NaIO_4_ and PEO/E8 composites within the broad range of considered temperature variation were obtained via a sum of the fitting functions from Equation (2) and Equation (3), expressed as:

(6) $$\varepsilon \prime \prime \left(f\right)=\frac{\sigma }{\varepsilon_{0}{\left(2\pi f\right)}^{n}}+F.T.\;\left\{\Delta \varepsilon_{KWW}\;\exp\left[-{\left(\frac{t}{\tau }\right)}^{\beta }\right]\right\}\;+\;\frac{\Delta \varepsilon_{HN}}{{\left[1+{\left(f/f_{0}\right)}^{a}\right]}^{b}}+\varepsilon_{\mathrm{der}}^{\prime \prime }$$

i.e., they were again obtained through the addition of the terms of the classical KWW and HN models. The physical meaning of the parameters in Equation (6) is the same as above. For instance, Figure 20 represents the fits for the PEO/E8 composite. Compared to the previous case of use only Equation (3), the expression in Equation (6) better accounted for the thermo-induced dielectric changes in the polymer-LC composites, and the quality of the fits on *ε*″(*f*) spectra at higher temperatures became more gradually improved across the *T_g_*-region (Figure 21). Regarding the temperature behaviors of the best-fit parameters, the trends and the temperature thresholds were nearly the same as discussed beforehand. Examples are given in Figure 22. Hence, despite its complexity, the combined model, when applying Equation (6), appeared to be profitable for modeling the dielectric loss of the coupled molecular dipolar formations of PEO-E8LC in PEO/E8-based composites, and specifically for its complete analysis and interpretation in terms of dielectric relaxation behavior.

## 4. Conclusions

The dielectric relaxation in the PEO/E8 and PEO/E8/NaIO_4_ polymer/LC composites/complexes was investigated on a temperature scale in the range of their glass transition temperature (*T_g_*). The results from the analyses of the thermally induced changes of the frequency spectra of both impedance and dielectric permittivity were interrelated. The temperature dependencies of the studied characteristics followed the temperature phase changes of the composites at their *T_g_*. Both impedance and dielectric permittivity exhibited dielectric relaxation behaviors that were attributed to dipolar media. Due to the polymer softening above *T_g_*, the dielectric polarization, the ionic and AC conductivity of the studied composites were strongly enhanced, and the dielectric relaxation time became shorter.

Physical information about the dipolar molecular organization, dipole polarization, and dipole relaxation (dielectric relaxation of dipoles), which was necessary to elucidate the hypothesis on the possible polymer-LC intermolecular dipole structures formed between the polar polymer PEO and polar molecules of the nematic LC E8 in PEO/E8-based composites and complexes, was obtained. We specified an appropriate model for the characterization of the frequency spectra of the dielectric loss of these composites and their thermo-induced changes. Through our comparative study of the PEO/E8 and PEO/E8/NaIO_4_ composites (two dielectrics with the same compositional PEO:E8 ratio) under identical experimental conditions, the results from the analyses of complex electrical impedance and dielectric responses showed that the doping of ionic salt, which formed salt-complexed ion-dipolar structures, enhanced the dynamics of the relaxation processes.

The information obtained for the dielectric relaxation in the studied composites and its temperature behavior is significant for understanding the structures of such types of glass-forming polymer/LC composites and complexes, namely ion conductors. The results from this study are of practical significance in relation to the use of these composites and related systems as materials for applications in energy storage devices, flexible organic electronics, sensorics, and mechatronics. The results are also of importance when estimating the thermo-induced changes and the thermal stability around *T_g_* of corresponding devices based on thin films of the considered polymer-LC materials.

## Data Availability

The data presented in this study are available on request from the corresponding author.

## Supplementary Materials

The following are available online at https://www.mdpi.com/article/10.3390/polym13244465/s1, Figure S1: Molecular structures of LC E8 and PEO, Figure S2: Morphology of PEO/E8 and PEO/E8/NaIO_4_ composite films, Figure S3: DSC data, Figure S4: Impedance spectra of PEO, PEO/E8 and E8LC at room temperature, Figure S5: Example of impedance spectrum of PEO/E8/NaIO4, Figure S6: Spectrum of dielectric loss of PEO at room temperature.
