# Supplementary material for: Ion-Conducting Flexible Thin Films of Composites from Poly(ethylene oxide) and Nematic Liquid Crystals E8—Characterization by Impedance and Dielectric Relaxation Spectroscopy
Source: Polymers (Basel). 2021 Dec 20;13(24):4465. doi: 10.3390/polym13244465 (PMC8704298; doi:10.3390/polym13244465)
Supplement: Supplementary file 1 [file polymers-13-04465-s001.zip › polymers-1464895-supplementary.pdf]

polymers

MDPI

Supplementary

Ion-Conducting Flexible Thin Films of Composites from Poly(Ethylene Oxide) and Nematic Liquid Crystals E8—Characterization by Impedance and Dielectric Relaxation Spectroscopy

Georgi B. Hadjichristov [1,*], Todor E. Vlakhov [1], Yordan G. Marinov [1] and Nicola Scaramuzza [2]

1 Georgi Nadjakov Institute of Solid State Physics, Bulgarian Academy of Sciences, 72 Tzarigradsko Chaussee Blvd.,1784 Sofia, Bulgaria; todor_vlakhov@issp.bas.bg (T.E.V.); ymarinov@issp.bas.bg (Y.G.M.)
2 Dipartimento di Fisica, Università degli Studi della Calabria (UNICAL), Via P. Bucci, Cubo 33B, 87036 Rende, Italy; nicola.scaramuzza@fis.unical.it
* Correspondence: georgibh@issp.bas.bg

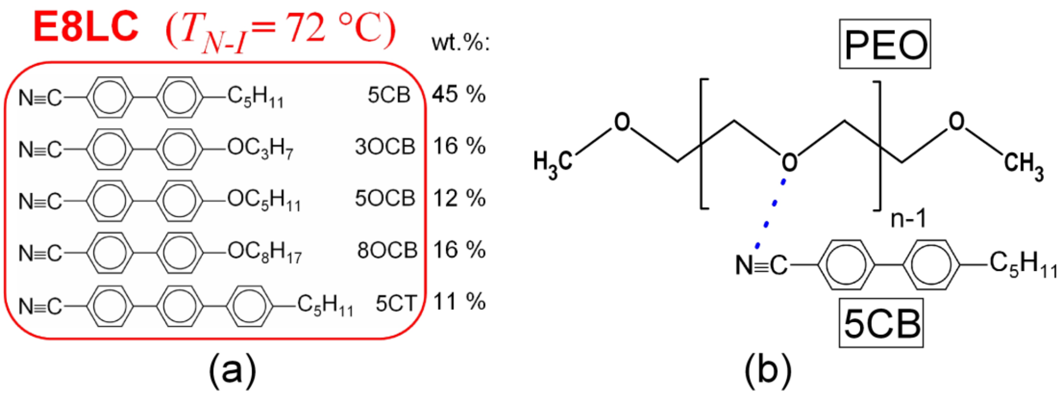

Figure S1. (a) Chemical structures of E8LC constituents; **(b)** intermolecular coupling of LC molecule (in particular 5CB) to PEO structure.

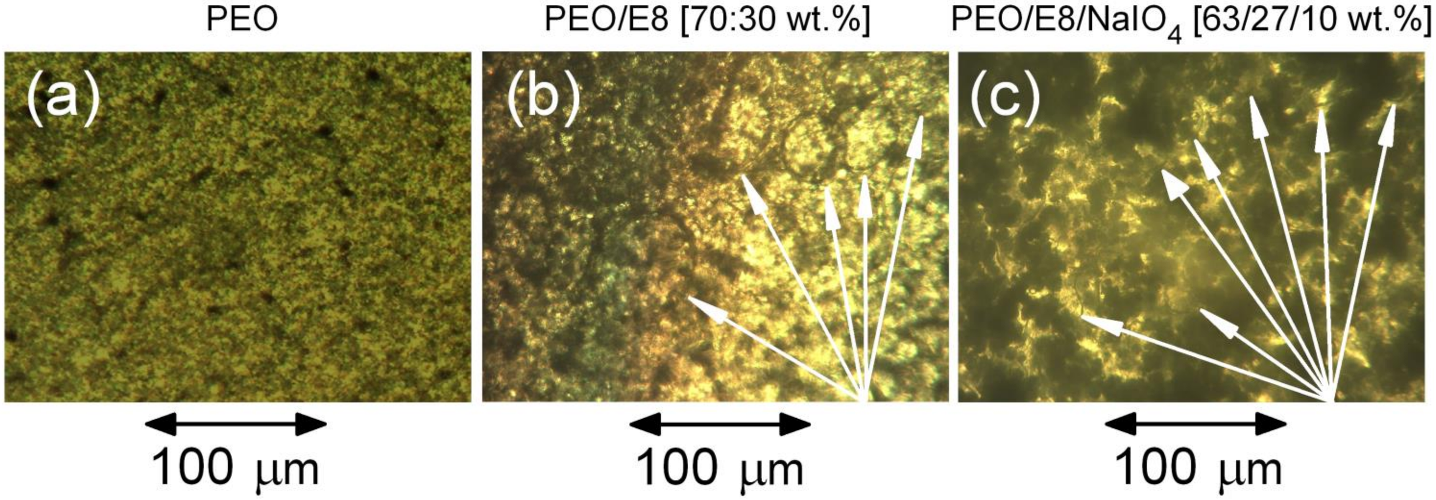

Figure S2. Optical micrographs taken at room temperature (25 °C) for 150 μm-thin films of PEO **(a)**, as well as of the studied PEO/E8 **(b)** and PEO/E8LC/$NaIO_4$ **(c)** composites. The films were between crossed analyzer and polarizer. The white arrows point out the shapes of some of the LC droplets on the microscope images.

By our supplementary studies in transmission by means of NU-2 universal polarizing microscope (Carl Zeiss Jena), the morphology of the PEO/E8 and PEO/E8LC/$NaIO_4$ composite films was thoroughly investigated, including also its dependence on the variation of the LC percentage and temperature. The results are published elsewhere [1–3]. Here we should only mention that the microstructure of these composite films is characterized with the presence of mixed polymer and LC phases. Distinct microdroplets from the included LC take also place within the PEO/E8 and PEO/E8LC/$NaIO_4$ films. Similar to the polymer dispersed liquid crystals, soft micro-sized E8LC droplets (relatively large) disposed in a matrix of the polymer PEO were formed (with oval shapes) by phase separation during the films preparation (Figure S2(b,c)).

1. Koduru, H.K.; Marinov, Y.G.; Scarpelli, F.; Hadjichristov, G.B.; Petrov, A.G.; Godbert, N.; Scaramuzza, N. Polyethylene oxide (PEO) - Liquid crystal (E8) composite electrolyte membranes: microstructural, electrical conductivity and dielectric studies. *J. Non-Cryst. Solids* **2018**, *499*, 107–116.
2. Koduru, H.K.; Marinov, Y.G.; Hadjichristov, G.B.; Scaramuzza, N. Characterization of polymer/liquid crystal composite based electrolyte membranes for sodium ion battery applications. *Solid State Ionics* **2019**, *335*, 86–96.
3. Hadjichristov, G.B.; Marinov, Y.G.; Ivanov, Tz.E.; Koduru, H.K.; Scaramuzza, N. PEO/E8 Polymer-Liquid Crystal Flexible Complex Blend Electrolyte System for Na Ions. In *Liquid and Single Crystals: Properties, Manufacturing and Uses*; Goosen, J., Ed.; Nova Science Publ.: New York, USA, 2020; pp. 1–64.

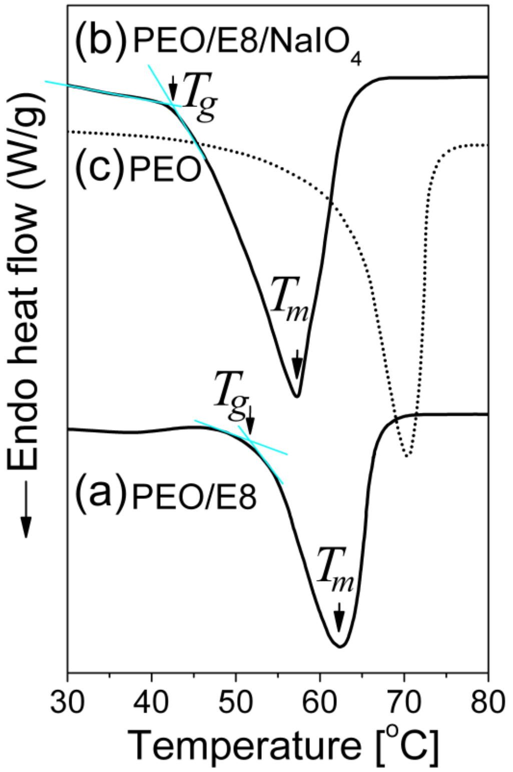

Figure S3. DSC thermograms obtained on heating of the studied composites: **(a)** PEO/E8; **(b)** PEO/E8/$NaIO_4$; **(c)** pure PEO. The arrows indicate the values of glass transition temperatures (T_g) and melting temperatures (T_m) of both composites. Experimental details for DSC studies with a scan rate of 10 °C/min are given in [1,2]. For each DSC curve, T_g and T_m of the samples were determined from the inflection point of the endothermic process curve and the center of the endothermic peak, respectively.

1. Koduru, H.K.; Marinov, Y.G.; Scarpelli, F.; Hadjichristov, G.B.; Petrov, A.G.; Godbert, N.; Scaramuzza, N. Polyethylene oxide (PEO) - Liquid crystal (E8) composite electrolyte membranes: microstructural, electrical conductivity and dielectric studies. *J. Non-Cryst. Solids* **2018**, *499*, 107–116.
2. Koduru, H.K.; Marinov, Y.G.; Hadjichristov, G.B.; Scaramuzza, N. Characterization of polymer/liquid crystal composite based electrolyte membranes for sodium ion battery applications. *Solid State Ionics* **2019**, *335*, 86–96

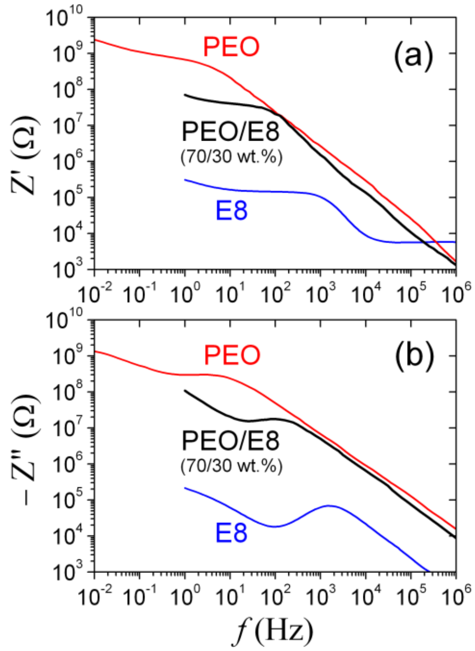

Figure S4. Comparison of frequency spectra of complex electrical impedance (Z^*) of thin films of PEO (150 μm thin), PEO/E8 (70/30 wt.%) composite (150 μm thin) and E8LC (25 μm thin): **(a)** real part (Z') and **(b)** imaginary part (Z''). The spectra were recorded under identical experimental conditions, at room temperature equal to 26 °C.

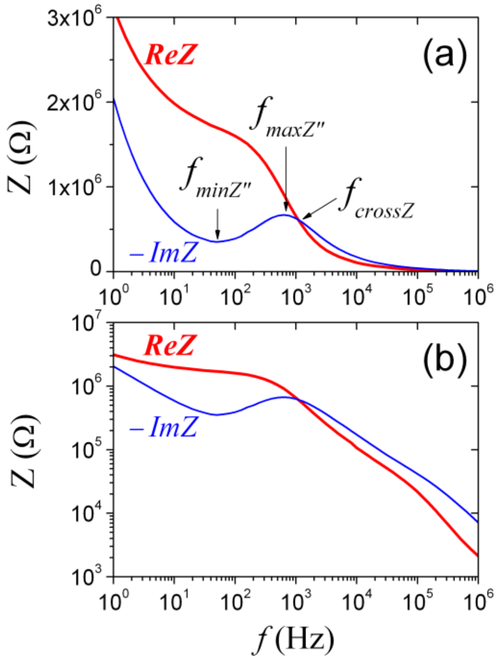

Figure S5. Representative {Z', Z''} pair of PEO/E8/$NaIO_4$ (recorded at 25 °C). The values of the frequencies $f_{maxZ''}$ and f_{crossZ} are indicated. Z data are presented in linear **(a)** and logarithmic **(b)** scales.

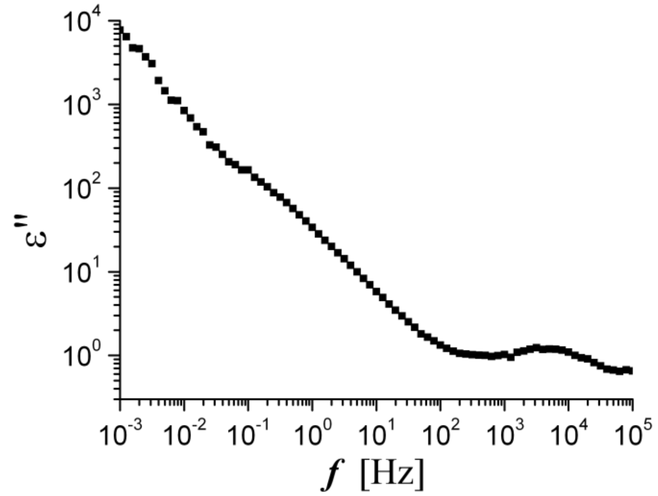

Figure S6. ε'' dielectric spectrum of pure PEO at room temperature. The peak at about 10 kHz corresponds to the polarization relaxation in this polymer.
